# Impact of CodY protein on metabolism, sporulation and virulence in *Clostridioides difficile* ribotype 027

**DOI:** 10.1371/journal.pone.0206896

**Published:** 2019-01-30

**Authors:** Nadine Daou, Yuanguo Wang, Vladimir M. Levdikov, Madhumitha Nandakumar, Jonathan Livny, Laurent Bouillaut, Elena Blagova, Keshan Zhang, Boris R. Belitsky, Kyu Rhee, Anthony J. Wilkinson, Xingmin Sun, Abraham L. Sonenshein

**Affiliations:** 1 Department of Molecular Biology and Microbiology, Tufts University School of Medicine, Boston, MA, United States of America; 2 Department of Infectious Disease and Global Health, Cummings School of Veterinary Medicine, Tufts University, North Grafton, MA, United States of America; 3 Structural Biology Laboratory, Department of Chemistry, University of York, York, United Kingdom; 4 Department of Medicine, Division of Infectious Diseases, Weill Cornell Medical College, New York, NY, United States of America; 5 Broad Institute of MIT and Harvard, Cambridge, MA, United States of America; 6 Department of Molecular Medicine, Morsani College of Medicine, University of South Florida, Tampa, FL, United States of America; Institut Pasteur, FRANCE

## Abstract

Toxin synthesis and endospore formation are two of the most critical factors that determine the outcome of infection by *Clostridioides difficile*. The two major toxins, TcdA and TcdB, are the principal factors causing damage to the host. Spores are the infectious form of *C*. *difficile*, permit survival of the bacterium during antibiotic treatment and are the predominant cell form that leads to recurrent infection. Toxin production and sporulation have their own specific mechanisms of regulation, but they share negative regulation by the global regulatory protein CodY. Determining the extent of such regulation and its detailed mechanism is important for understanding the linkage between two apparently independent biological phenomena and raises the possibility of creating new ways of limiting infection. The work described here shows that a *codY* null mutant of a hypervirulent (ribotype 027) strain is even more virulent than its parent in a mouse model of infection and that the mutant expresses most sporulation genes prematurely during exponential growth phase. Moreover, examining the expression patterns of mutants producing CodY proteins with different levels of residual activity revealed that expression of the toxin genes is dependent on total CodY inactivation, whereas most sporulation genes are turned on when CodY activity is only partially diminished. These results suggest that, in wild-type cells undergoing nutrient limitation, sporulation genes can be turned on before the toxin genes.

## Introduction

*Clostridioides difficile*, a spore-forming bacterial pathogen, is now the primary cause of antibiotic-associated diarrhea, an infection usually acquired during stays in healthcare facilities. At present, >500,000 patients in the USA are newly diagnosed with *C*. *difficile* infection (CDI) each year and 75,000–175,000 cases of recurrent CDI are seen, leading to an increase in healthcare costs of $4.2 billion [1–3]. While current treatments cure almost 90% of primary infections, recurrent infection is so high that thousands of patients are on long-term antibiotic treatment and more than 25,000 CDI patients die each year [1, 4]. The ability of the bacterium to be so effective in causing disease depends on two critical aspects of its biology. Pathogenic *C*. *difficile* produces at least two potent toxins, TcdA and TcdB, that cause major disruptions of the mammalian intestinal tract [5]. In addition, the bacteria form spores in the GI tract, a process that makes a significant fraction of the infecting cells resistant to all antibiotics and provides a way for this oxygen-sensitive anaerobe to survive in the environment. Spore formation appears to be the primary factor responsible for the unusual frequency of recurrent *C*. *difficile* infection; the spores released from the GI tract are difficult to kill, survive in the environment and are able to cause a new round of infection in patients who have completed their antibiotic treatment. Discovering factors that influence toxin production or spore formation or both is critical to our understanding of infection and has the potential to lead to novel ways of preventing and treating *C*. *difficile* infection.

The genes that encode the toxins have no known impact on sporulation. Similarly, most sporulation proteins are not known to influence toxin synthesis directly. (There is some evidence that the sporulation regulator Spo0A contributes to toxin gene regulation, but the effect varies from strain to strain [6–8]). The toxin genes are transcribed at very high levels by RNA polymerase containing the toxin gene-specific sigma factor TcdR [9]. Surprisingly, a null mutation in *tcdR* causes a decrease in sporulation efficiency and the heat-resistance of the resulting spores [10], implying that one or more genes normally transcribed by TcdR-containing RNA polymerase encode proteins that affect spore formation. The conditions that lead to TcdR activity are complex. During exponential growth in rich medium, the *tcdR* gene is transcribed at low levels by σ^A^-containing RNA polymerase, the primary form of RNA polymerase in growing cells. When cells reach stationary phase, the *tcdR* gene is much more highly expressed, first from the σ^A^ -dependent promoter and then, as TcdR accumulates, from a TcdR-dependent promoter [11, 12]. A third sigma factor, σ^D^ (the motility sigma factor), also contributes to *tcdR* gene expression [13]. When TcdR accumulates, it directs RNA polymerase to the promoters of the toxin genes, leading to high-level toxin gene expression [9].

Although they may or may not influence each other directly, both sporulation and toxin synthesis are turned on when cells experience nutrient limitation, e.g., when cells in laboratory culture in a complex medium reach stationary phase. At least two global regulatory proteins are known to contribute to this form of regulation. CcpA, the regulatory protein that is activated when rapidly metabolizable sugars are in excess, is a repressor of the *tcdR* and toxin genes and represses sporulation as well [14]. The global regulator, CodY, is also a strong repressor of the *tcdR* gene [15, 16], as well as an inhibitor of sporulation [17]. Nawrocki *et al*. [17] found that in the ribotype 027 strain UK1, a null mutation in the *codY* gene increases spore formation 1000-fold in cells growing in 70:30 medium. CodY is found in nearly all low G+C Gram-positive bacteria and is activated by binding of one of the branched-chain amino acids (BCAAs; isoleucine, leucine, valine) [18] and, in most species, GTP [19–23]. The responsiveness of CodY to BCAAs implies that the cell has evolved to assess accumulation of these amino acids as a major indicator of nutrient availability; a likely reason is that the BCAAs are not only used for protein synthesis but are also the precursors of branched-chain fatty acids, the principal membrane fatty acids of most Gram-positive bacteria [24, 25]. The GTP level in the cell influences the ability to synthesize RNA, but is also a general indicator of amino acid availability; when the intracellular concentration of any amino acid drops below the level needed for its incorporation into protein, the GTP pool drops significantly due to the conversion of GTP to pppGpp via the stringent response [26, 27]. Thus, when certain nutrients become limiting, both toxin and sporulation genes can potentially be turned on.

The CodY protein represses transcription of hundreds of genes and activates transcription of dozens of other genes in many Gram-positive species [15, 16, 28–43]. The direct target genes tend to be involved in multiple aspects of nutrient uptake, metabolism and virulence; some target genes encode other regulators that are the direct activators or repressors of the apparent CodY targets. A previous microarray analysis of genes whose expression is altered by a *codY* null mutation in the *C*. *difficile* ribtoype 012 strain JIR8074 (also known as 630E) identified 146 genes that are negatively regulated >4-fold by CodY and 19 genes that are positively regulated [15].

The diversity and complexity of the CodY regulon raises important issues about how and why the cell uses such a regulator to control multiple, seemingly unrelated processes. A key aspect of our approach was to subject a panel of CodY point mutants with different levels of residual activity to genome-wide transcription analysis by RNA-seq in order to identify genes that are regulated at different levels of CodY activity. Similar approaches have been used to analyze the CodY regulons in *Bacillus subtilis* [30] and *Staphylococcus aureus* [42]. This type of analysis provides a global picture of *C*. *difficile*’s strategy for altering virulence and sporulation properties in response to various levels of nutrient availability.

## Results

### Virulence of *codY* mutant strains

**Fig 1:**
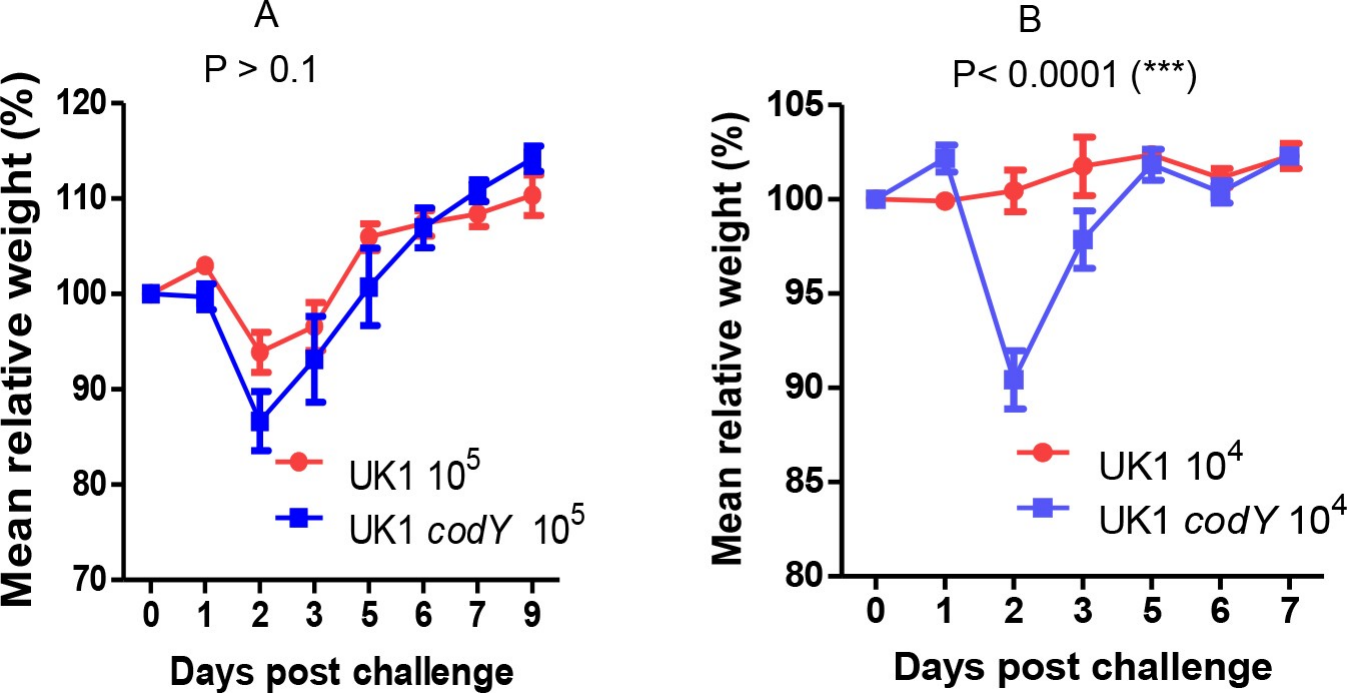
Infection outcomes induced by strain UK1 and its *codY* mutant derivative. Groups of mice (n = 10) were infected with spores of strain UK1 and its *codY* null mutant (UK1 *codY*, also known as LB-CD16) and were monitored for weight change at doses of 10^5^ spores per mouse (A) or 10^4^ spores per mouse (B). Weight changes between the two groups were analyzed by 2-way ANOVA. The p values were calculated based on the differences in weight changes between the mice infected with the wild-type strain and the mice infected with the codY null mutant over the entire time course of the experiment (days 0–9 for Fig 1A and days 0–7 for Fig 1B). The impact of these infections on mouse diarrhea and viability is listed in Table 1.

The documented role of CodY as a repressor of *C*. *difficile* toxin gene expression in laboratory cultures [15, 16] raised the possibility that a *codY* null mutant strain might overexpress the toxin genes during infection and therefore be hypervirulent. To address this possibility, we tested the virulence of strain LB-CD16, a *codY* null mutant [44] of strain UK1, a ribotype 027 isolate from the Stoke-Mandeville outbreak of 2003 [45]. (The mutation was created by insertion within *codY* of an intron containing an erythromycin-resistance determinant and is noted as *codY*::*intron*::*erm*.) In a mouse model of infection in which mice are fed a cocktail of antibiotics before infection [46], strain UK1 and the *codY* null mutant (LB-CD16) induced diarrhea and weight loss in all mice infected at a dose of 10^5^ spores per mouse (Fig 1A and Table 1); 10% of the mice died after infection by UK1, 20% after infection by the *codY* null mutant. At a dose of 10^4^ spores per mouse, strain UK1 was greatly reduced in virulence, but the *codY* null mutant caused diarrhea and loss of weight (without death) in nearly all infected mice (Fig 1B and Table 1).

**Table 1:** Diarrhea in mice infected with *codY*^+^ and *codY* null mutant strains.

Strain	Dose of spores per mouse	Mice with diarrhea (%)	Death of infected mice (%)
UK1	10^5^	100	10
UK1 *codY*	10^5^	100	20
UK1	10^4^	20	0
UK1 *codY*	10^4^	90	0

Groups of 10 mice pretreated with antibiotics and infected with the indicated doses of spores of *C*. *difficile* strains UK1 and UK1 *codY* (LB-CD16) were monitored for survival and the occurrence of diarrheal symptoms over the course of 7 days of infection.

### Toxin titers in parental and mutant strains

**Fig 2:**
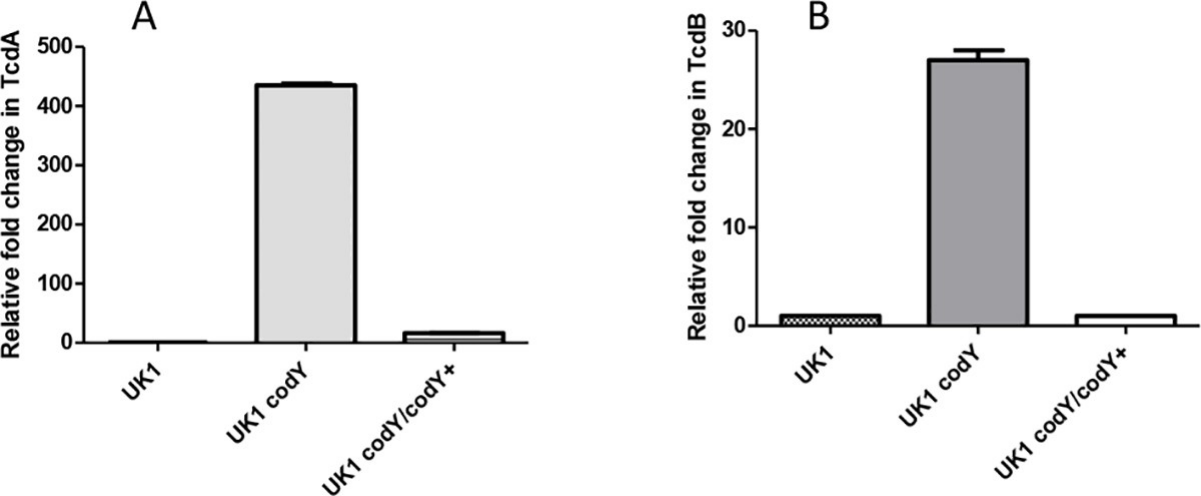
Toxin titers in cultures of parent and *codY* null mutant strains. **R**elative levels of TcdA and TcdB in culture supernatants of UK1, UK1 *codY* (LB-CD16) and UK1 *codY/codY*^*+*^ (ND-CD10) collected after 24 hrs of bacterial growth were determined by ELISA. Two samples were assayed for each toxin for each strain; toxin levels were averaged and normalized to the values in the wild-type strain (UK1) set at 1.0. Error bars were created for all pairs of samples, but for some pairs the difference was so small that the bars are not visible.

**Fig 3:**
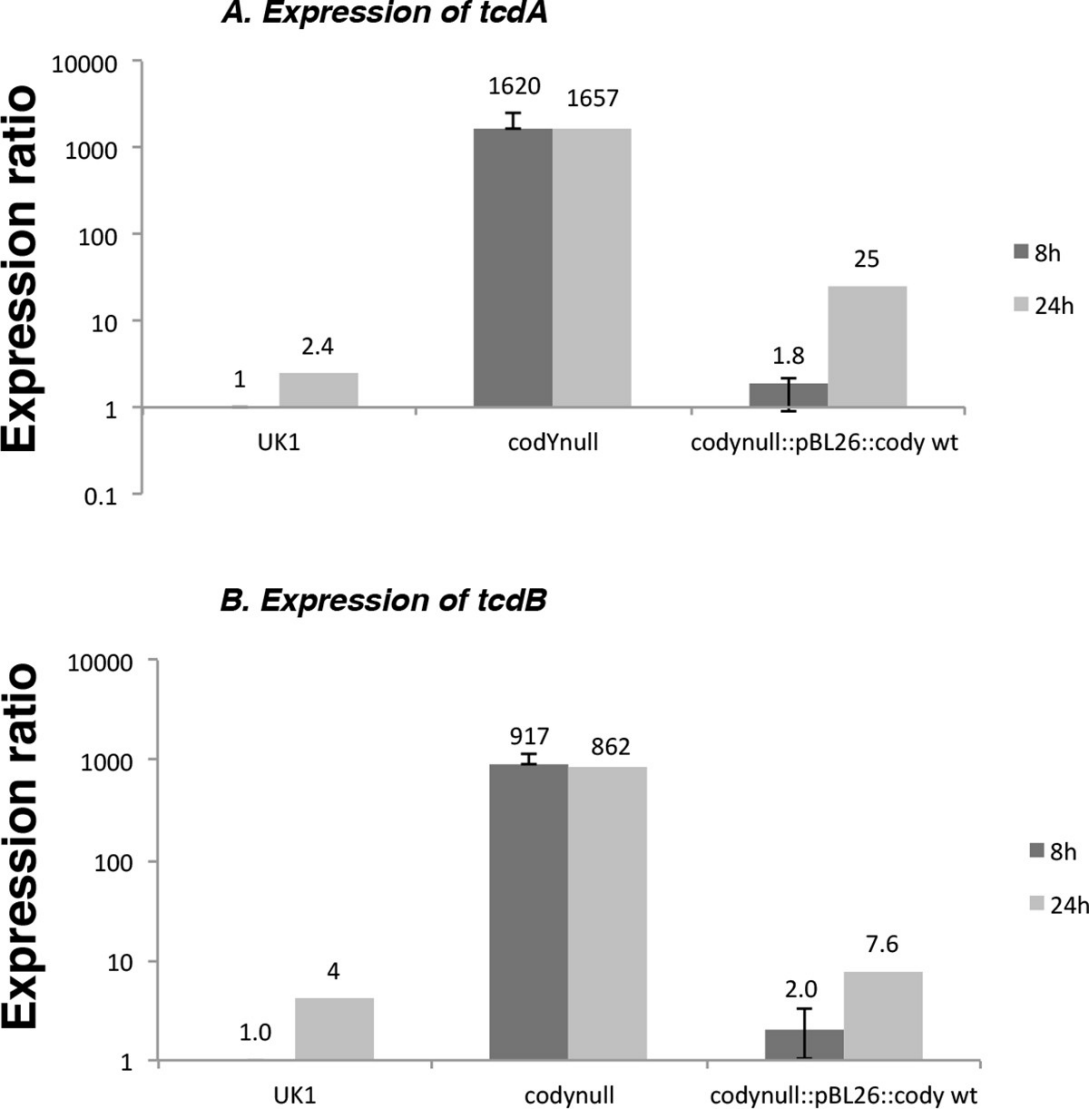
Effect of a *codY* null mutation on *tcdA* and *tcdB* transcription in strain UK1. Cultures of strains UK1, LB-CD16 (*codY*::*intron*::*erm*) and ND-CD10 (*codY*::*intron*::*erm codY*^*+*^) were grown in CDMM medium and samples were removed at 8 hrs (late exponential growth phase) and 24 hrs (stationary phase). RNA was extracted and assayed for *tcdA* and *tcdB* expression by qRT-PCR (see Materials and Methods). Results for the toxin genes were normalized to those obtained for the *rpoA* gene.

To test the relationship between the hypervirulence of strain LB-CD16 (UK1 *codY*) and toxin titer, culture fluids of laboratory cultures that had grown for 24 hrs at 37°C in an anaerobic chamber were tested for toxin titers by ELISA assays. As shown in Fig 2, the culture fluid of the *codY* null mutant contained considerably more TcdA (Toxin A) and TcdB (Toxin B) than did its parent strain. These results were consistent with the much higher level of expression of the toxin genes in laboratory cultures of strain UK1 *codY* (Fig 3).

To verify that toxin overproduction in strain LB-CD16 was a result of the *codY* null mutation, complementation was achieved by integrating a wild-type copy of *codY* contained within the transposon Tn*916* at a non-homologous locus in the *C*. *difficile* chromosome. As shown in Fig 2, the complemented strain had near-parental levels of toxin proteins. In addition, the vast overexpression of the *tcdA* and *tcdB* genes in the UK1 *codY* mutant strain was greatly reduced in the complemented strain (Fig 3).

### Creation of a family of CodY point mutants with different levels of residual activity

Whereas complete inactivation of CodY in strain UK1 led to about a 1000-fold increase in toxin gene expression at the onset of stationary phase (Fig 3), the level of CodY activity needed to repress the toxin genes has not been determined. To address this question and simultaneously determine the extent to which other CodY target genes are affected by partial inactivation of CodY in strain UK1, we created a family of *codY* point mutants with different levels of residual activity. We then determined the impact of these mutations on gene expression during mid-exponential phase in cells growing in a rich medium. Doing so prevented the potential contribution of changes in nutrient availability that would alter multiple other regulatory mechanisms that are likely to affect many of the CodY-regulated genes.

Our approach was to introduce mutations in the region of CodY that includes the BCAA-binding pocket in order to reduce, but not eliminate, the affinity of CodY for the BCAAs or the extent of CodY conformational change upon BCAA binding. To know which amino acid residues of CodY are likely to play a major role in interaction with and response to BCAAs, we determined the crystal structure of the N-terminal half of *C*. *difficile* CodY (CdCodY).

#### a) Structure of the GAF domain of CdCodY

**Fig 4:**
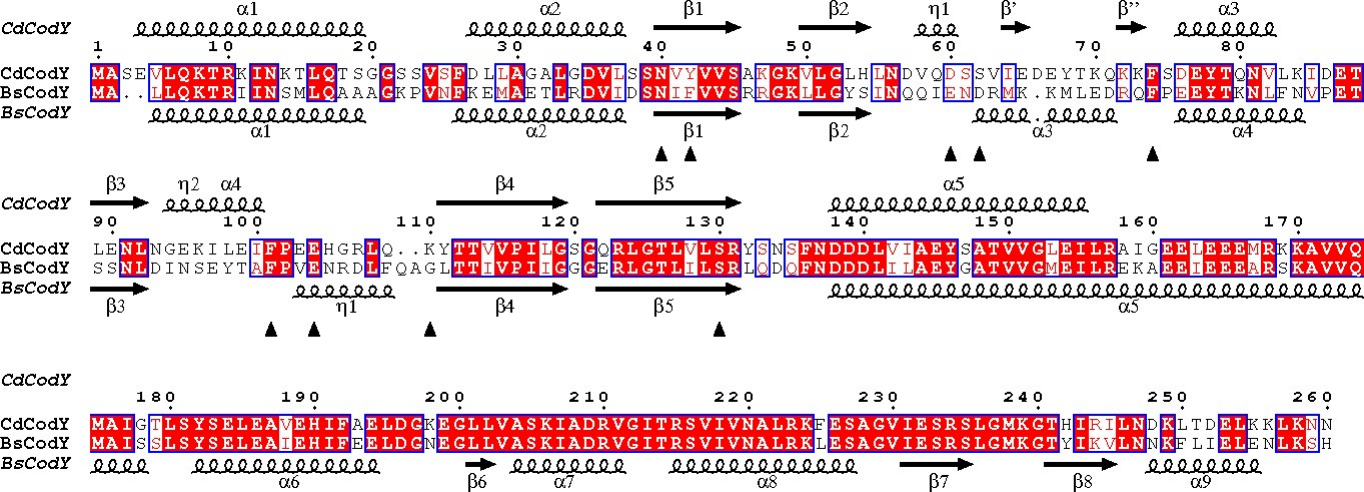
Alignment of the sequences of CodY from *C*. *difficile* (Cd) and *B*. *subtilis* (Bs). The protein secondary structures for the full-length CdCodY and BsCodY are depicted above and below the sequences, respectively. Sequence identities are indicated by red shading. Residues that form prominent interactions with the effector molecule (isoleucine) in *C*. *difficile* CodY are labeled with black arrowheads.

**Fig 5:**
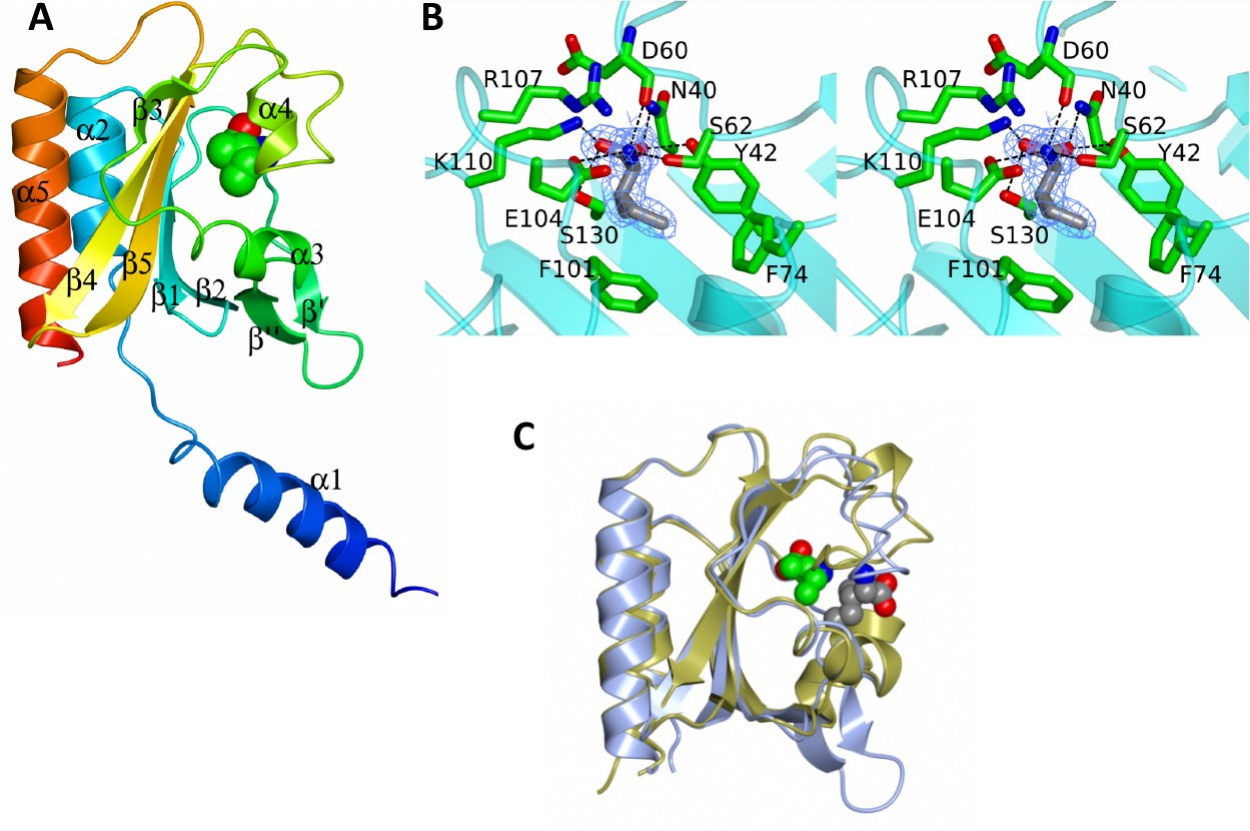
Structure of the GAF domain of *C*. *difficile* CodY. **A.** Ribbon tracing of chain A color ramped from the N-terminus (blue) to the C-terminus (red). The secondary structure elements are labeled. The isoleucine cofactor is shown as spheres and colored by atom: carbon, green; nitrogen, blue; oxygen, red. **B.** Stereo view of the ligand binding site with the isoleucine effector shown in cylinder format and with carbon atoms colored grey. 2F_o_-F_c_ electron density associated with the effector in the refined structure is contoured at 1σ and shown in light blue. Surrounding protein residues are shown in cylinder format with their carbon atoms colored in green. **C**. Comparison of the GAF domains of CodY from *C*. *difficile* and *B*. *subtilis* shown as blue and gold ribbons respectively with the isoleucine ligands shown as spheres with grey and green carbon atoms respectively. The different positions and orientations of the effector molecules are apparent.

The cGMP-specific phosphodiesterases- adenylyl cyclases-FhlA (GAF) domain of *B*. *subtilis* CodY (BsCodY) protein, formed by the N-terminal 155 residues, includes a loop of amino acid residues that interact with BCAAs; such binding induces a change in protein structure leading to increased affinity for CodY-binding sites [47–49]. The GAF domain sequences of *C*. *difficile* and *B*. *subtilis* CodY proteins (44% identity) are not nearly as similar as the C-terminal, DNA-binding domains (93% identity) (Fig 4). The crystal structure of the GAF domain of CdCodY bound to isoleucine was solved by molecular replacement and refined against data extending to 1.7 Å resolution, as described in Supplementary Materials and Methods and S1 Table. The CdCodY GAF domain was found to consist of a central, five-stranded, anti-parallel β-pleated sheet (β3-β4-β5-β1-β2) onto the bottom face of which are packed two α-helices, α2 and α4. Extending from the opposite face of the sheet are two extended loops; β2-β3 meanders across the top of the sheet forming a prominent β-hairpin and a short α-helix, whereas β3-β4 does not contain a regular secondary structure. The N-terminal α1 helix protrudes strikingly away from the rest of the GAF domain in CdCodY (Fig 5A). Although they differ in sequence, the tertiary structure of the CdCodY GAF domain is similar to that of the GAF domain of BsCodY (Fig 5C); 120 Cα atoms can be superposed with a root mean squared positional displacement (rmsΔ) of 1.5 Å.

In both CdCodY and BsCodY, the effector isoleucine is bound in a pocket on the face of the β-sheet that is distal to the long helices α2 and α5, but the positions and modes of isoleucine binding are quite different (Fig 5C). The CdCodY binding site is formed by residues from strands β1 and β5 from the β-pleated sheet and from the β2-β3 and β3-β4 segments of the polypeptide, which embrace the ligand so that it is completely enclosed in the protein interior (Fig 5B). Isoleucine is expected to bind to the protein as a zwitterion. The first of the carboxylate oxygens of isoleucine forms an ion-pairing interaction with the ε-NH_3_^+^ of K110 and a charge-dipole interaction with the side chain hydroxyl of S130, with the second carboxylate oxygen forming charge-dipole hydrogen bonds with the phenolic hydroxyl of Y42 and the side chain amide–NH_2_ of N40 (Fig 5B). Meanwhile the α-amino group of the ligand forms an ion-pairing interaction with the side chain carboxylate of E104 and charge-dipole interactions with the hydroxyl of the side chain of S62 and the main chain carbonyl of D60. As shown in Fig 5B, R107 plays an important role in ligand binding since its guanidino moiety forms a two-pronged ion pairing interaction with the side chain of E104. Collectively these interactions fulfill the electrostatic and hydrogen bonding potential of the ligand. The isobutyl side chain, which distinguishes BCAAs from other amino acids, projects into a pocket circumscribed by the aromatic side chains of Y42, F74 and F101, and the aliphatic portions of the side chains of S62, S130 and E104.

In the GAF domain of CdCodY, the isoleucine carboxylate abuts the β-sheet, while in BsCodY it is displaced from the sheet and oriented away from it such that the respective carboxylate carbon atoms are separated by 9 Å in Fig 5C. This structural comparison helps to explain why residues participating in BCAA binding in BsCodY are strongly conserved in the CodY orthologs of some other low G+C Gram-positive bacteria, but not in CodY from *C*. *difficile* (Fig 4**)**.

#### b) Creation of point mutations in the GAF domain

To create relatively conservative mutations in the *codY* gene based on the structure determined by crystallography, we modified some amino acid residues, such as F74 and F101, that interact directly with isoleucine, and others, such as E99, P102 and E103, that are nearby, but do not interact directly. Because of the difficulty in creating point mutations in a specific gene in the *C*. *difficile* UK1 chromosome, we cloned the wild-type or mutant genes within the conjugative transposon Tn*916*; when introduced by conjugation into *C*. *difficile* strain LB-CD16 (*codY*::*intron*::*erm*), the modified transposon inserted at random sites. In no case was the *codY*::*intron*::*erm* mutation altered. Instead the transconjugants have two or more copies of *codY*, one at the normal *codY* locus interrupted by an *erm*-containing intron and at least one additional copy (with or without a GAF domain point mutation) elsewhere on the chromosome. Analysis by qPCR allowed us to choose strains in which the lowest number of copies of Tn*916* carrying *codY* had integrated (S2 Fig). The presence of the expected point mutations was verified by amplifying and sequencing the versions of *codY* located within Tn*916*. The chromosomal locations of the transposons were also determined by sequencing (S2 Table); isolates in which the insertion occurred within a gene were removed from analysis.

### Altered gene expression in the panel of *codY* mutant strains

**Fig 6:**
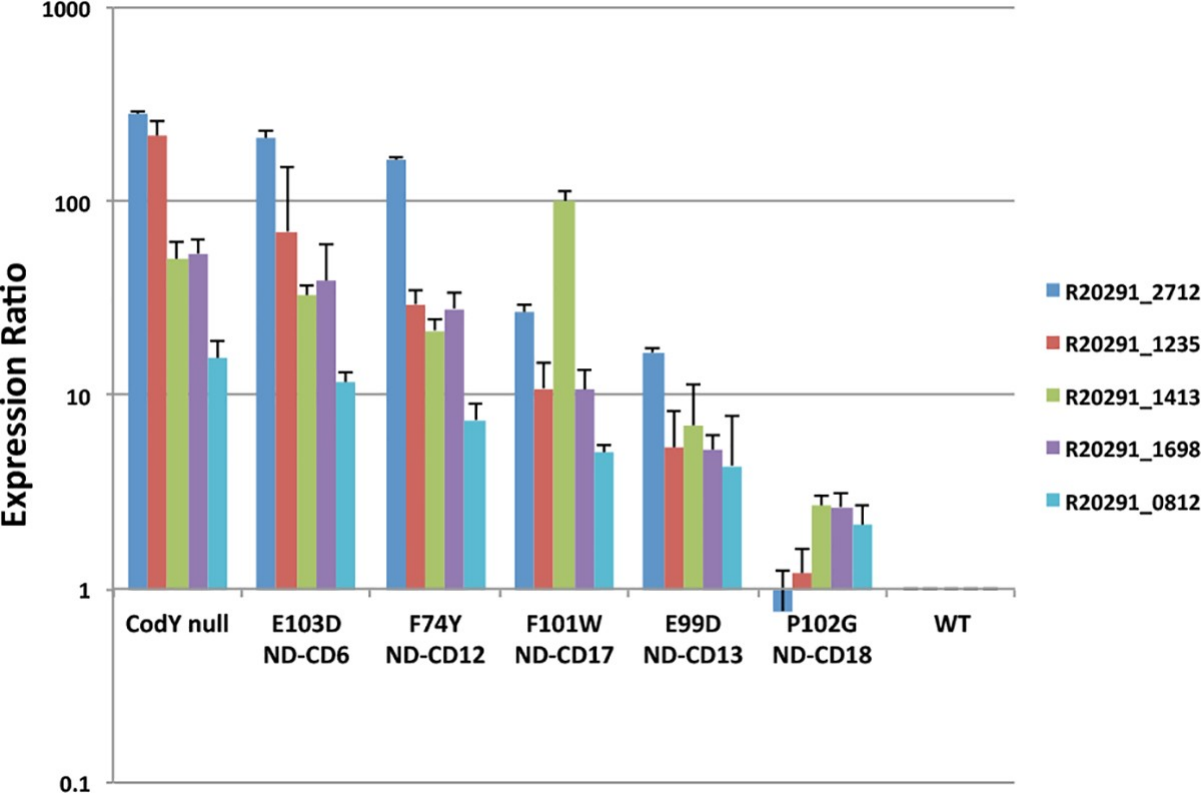
Effects of *codY* point mutations on expression of five *C*. *difficile* genes. Three-to-four individual cultures of wild-type strain UK1, its *codY* null mutant and five different *codY* point mutant strains were grown in TY medium to an OD_600_ = 0.4–0.6. RNA was extracted and assayed by qRT-PCR for the potential target genes R20291_2712 (encoding a peptidase), R20291_1235 (encoding chloromuconate cycloisomerase), R20291_1413 (*ilvC*), R20291_1698 (encoding a cell surface protein) and R20291_ 0812 (*glgC*). Counts were normalized to those for the *rpoA* gene and presented relative to the counts obtained with the wild-type strain.

To assess the effects of the various point mutations on CodY activity, RNA was extracted from cells grown in tryptose-yeast extract (TY) medium to mid-exponential phase (A_600_ = 0.4–0.6). After removal of DNA, the RNA was assayed for five specific transcripts by qRT-PCR. The genes were chosen based on previous microarray analysis of CodY-regulated genes in strain JIR8094 [15]. The results, shown in Fig 6, indicate that the five genes, all of which are repressed by CodY, were derepressed to different extents in the panel of mutants, suggesting that the mutants had different levels of residual CodY activity.

As shown in S3 Fig, all strains used for the experiments below (except for the original null mutant) produced significant levels of CodY protein. Unlike the case in *B*. *subtilis*, however, the *codY* gene of *C*. *difficile* is repressed by CodY. As a result, the concentration of CodY protein in most or all point mutants was higher than in wild-type cells (S3 Fig). Thus, the extent of gene regulation mediated by various point mutants is a complex result of both the mutant protein’s intrinsic activity and the extent to which it is overexpressed.

Based on their apparent intermediate levels of residual CodY activity, RNA from the wild-type strain, the *codY*::*intron*::*erm* null mutant and three of the point mutant strains (ND-CD12 *codY*::*intron*::*erm codY* [F74Y]; ND-CD13 *codY*::*intron*::*erm codY* [E99D] and ND-CD17 *codY*::*intron*::*erm codY* [F101W]) was then subjected to RNA-seq analysis. Of the 3505 protein-encoding genes annotated for this strain, DESeq analysis [50] identified 522 and 79 genes with more than a 3-fold higher or lower transcript abundance, respectively, and with an adjusted P value <0.05 in the null mutant compared to the parent strain. Transcript levels in all strains were then converted to RPKMO values (reads per kb of gene length per million reads aligning to all annotated ORFs in sample) [51] (see S4 Table for all values obtained). To further reduce the likelihood of erroneous identification of CodY-regulated genes, we excluded genes whose highest average normalized transcript level was less than 2 RPKMO in either the wild-type strain (for positively regulated genes) or the *codY* null mutant strain (for negatively regulated genes) and genes whose RPKMO value for one of the null mutant samples was within the range of wild-type samples. This filtering reduced the numbers of genes over- and underexpressed in the *codY* null mutant to 495 and 57, respectively (S3 Table). These genes may not represent the entire CodY regulon, since the exclusion of genes whose transcript level was affected less than 3-fold by a *codY* null mutation is arbitrary and is likely to exclude some genes whose expression is managed by other proteins in addition to CodY (see below). The impact of the three point mutations on gene expression as measured by RNA-seq was similar to that seen by qRT-PCR (Fig 6) for the five genes tested using both analyses (see S3–S7 Tables for RNA-seq data for the genes in Fig 6).

The derepressed genes (S4A Table) included more than 200 nutrient transport and metabolism genes (including amino acid biosynthesis genes), 14 peptidase and protease genes, 12 regulatory proteins, 47 known sporulation genes, the genes of the major toxin locus (*tcdR tcdBEA*) and many genes of unknown function. The most highly underexpressed genes in the *codY* null mutant were the glucitol-sorbitol metabolism operon, pyruvate/formate metabolism genes and cysteine metabolism genes (S4B Table).

**Fig 7:**
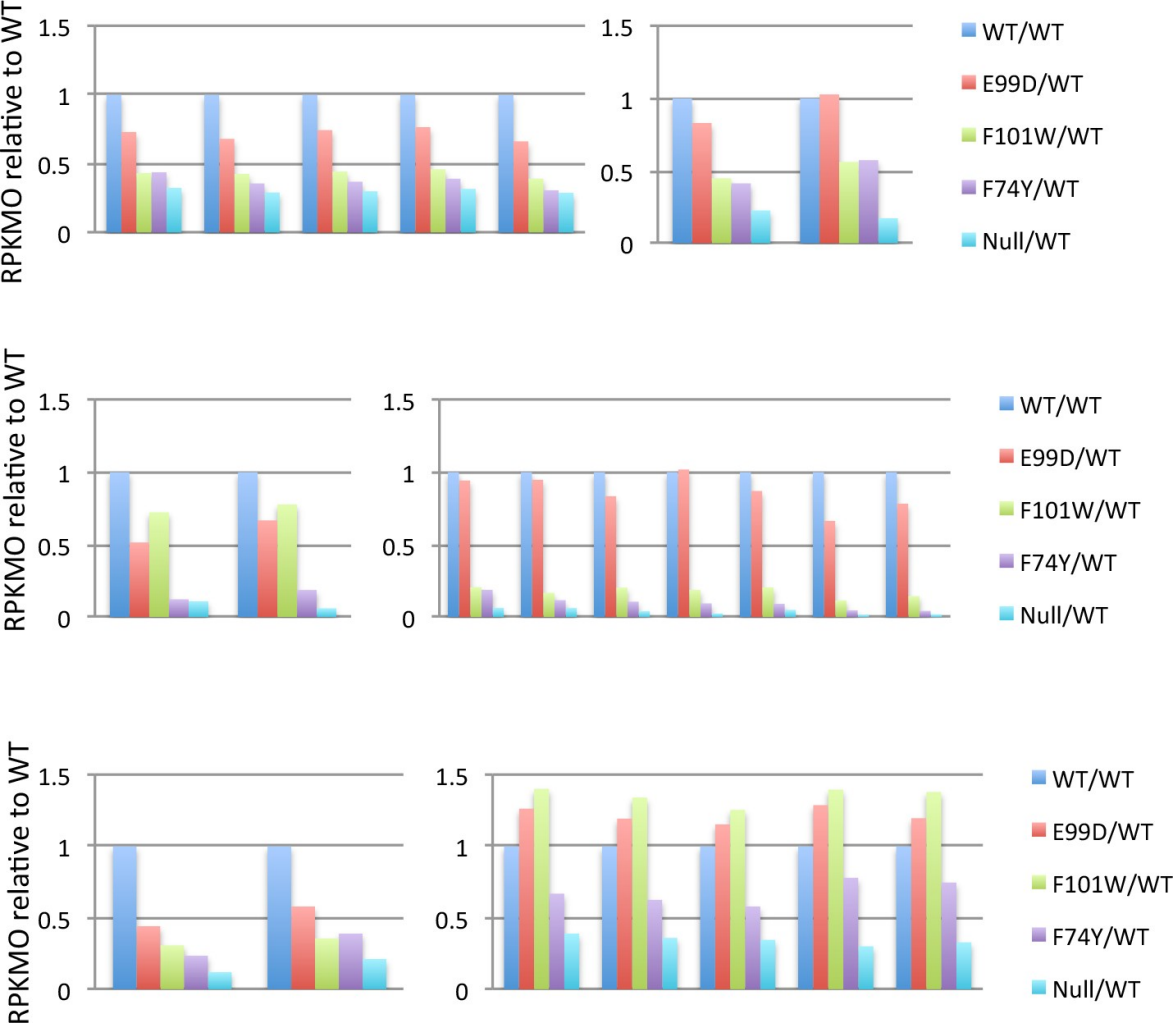
RNA-seq analysis of genes and operons positively regulated by CodY. Transcript levels (RPKMO values relative to wild-type, which was set at 1.0) in the *codY* null mutant and each of three point mutants are shown for examples of the 57 genes whose transcript levels were >3-fold lower in the *codY* null mutant than in the wild-type strain. The different colored patterns for each gene indicate the relative transcript levels in each of the mutant strains. Sections A-F present the results for all the genes of six different clusters. In clusters A and B, the gene names are their numbers in the R20291 genome.

**Fig 8:**
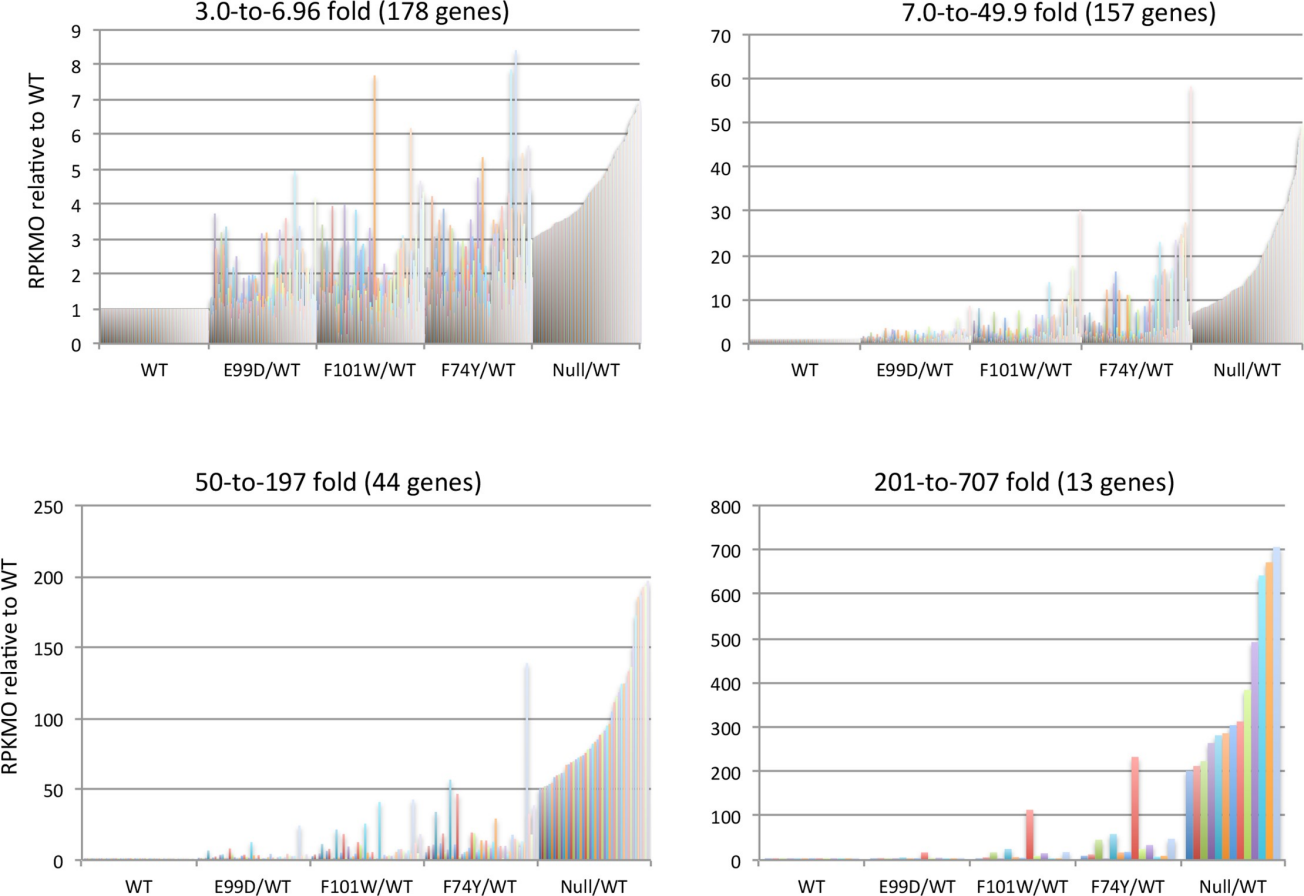
RNA-seq analysis of genes negatively regulated by CodY. Of the 495 genes whose transcript levels (measured as RPKMO) were >3-fold higher in the *codY* null mutant than in the wild-type strain, the relative levels for 392 genes are shown for the null mutant and three point mutants. The genes were divided into subgroups according to the level of overexpression in the null mutant.

**Fig 9:**
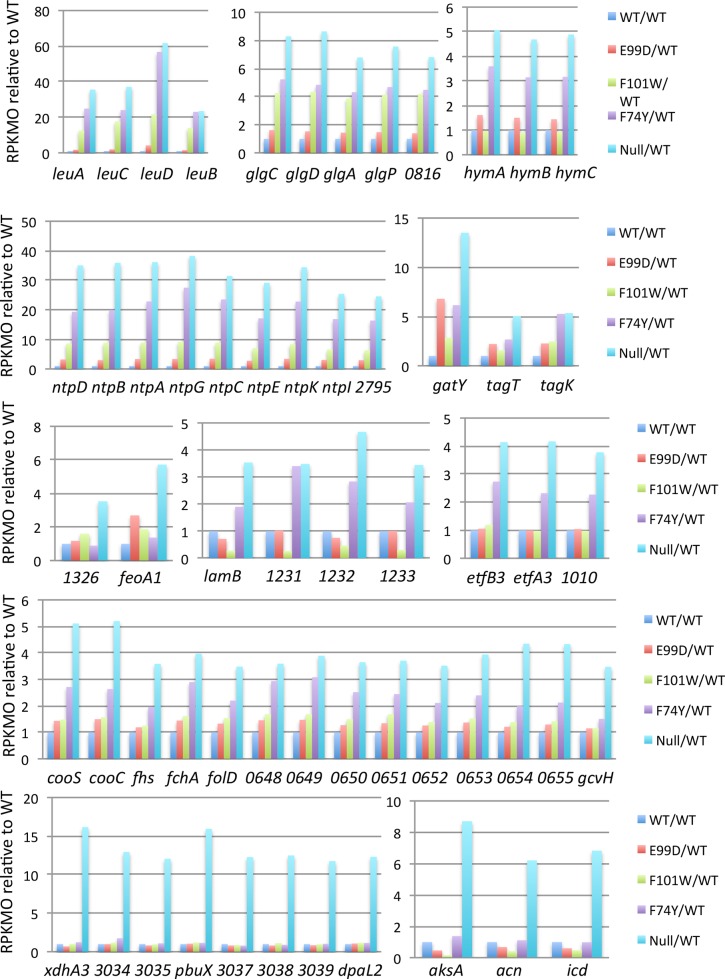
RNA-seq analysis of individual metabolic operons negatively regulated by CodY. Examples of the effects of the *codY* null mutation and three point mutations on individual negatively regulated operons are shown. For each gene, the average RPKMO value for wild-type cells was assigned a value of 1.0 and average transcript levels in each mutant strain were calculated relative to that in the wild-type. The different colored patterns for each gene indicate the relative transcript levels in each of the mutant strains. Each of the A-K parts of the figure present separate gene clusters.

When the transcript levels of both negatively and positively regulated CodY target genes were compared in the panel of point mutants, the overall pattern was consistent with the assumption that the strain expressing the E99D mutant form of CodY is closest to the wild-type for both negative and positive regulation, that the strain expressing the F74Y mutant form is closest to the null mutant (despite its own high level of expression, S3 Fig), and that the strain expressing the F101W mutant form has intermediate residual activity. This pattern can be seen in Fig 7 for examples of the 57 positively regulated genes and in Table 2 and Figs 8 and 9 for the negatively regulated genes. The full sets of positively and negatively regulated genes for the panel of *codY* mutants can be found in S4–S7 Tables.

**Table 2:** Overexpressed genes in *codY* mutant strains.

Overexpression compared to WT	*codY* mutation			
	Null	F74Y	F101W	E99D
>3-fold	495	257	173	98
>5-fold	333	149	105	39
>10-fold	210	91	57	26
>20-fold	135	43	40	20
>50-fold	71	17	28	15
>100-fold	40	7	23	14

As indicated in the first line of data, 495 genes were overexpressed >3-fold in the *codY* null mutant compared to the WT. Of those 495 genes, 333 were overexpressed >5-fold, 210 were overexpressed >10-fold, etc. Members of the group of genes overexpressed in the null mutant were overexpressed to various extents in the individual point mutants. The ethanolamine gene cluster (CDR20291_1828–1846) has such a low level of expression in the *codY* null mutant that 13 of the 19 genes were excluded from the listing. However, this gene cluster was very highly overexpressed in the point mutants. The pattern of expression is presented in detailbelow.

The transcript levels of some genes positively regulated by CodY were significantly reduced in all of the point mutants, whereas the transcript levels of other genes were reduced in only one or two of the point mutants (Fig 7), suggesting that for some genes the mutant forms of CodY are still able to serve as activators of transcription.

Similarly, the transcript levels of some genes negatively regulated by CodY were markedly increased in one or two or three point mutants whereas the transcript levels of others were not significantly elevated in any of the point mutants (Fig 9). The similarity in the patterns of transcript abundance seen for all the genes of a given operon argues that these patterns are a true indication of the specific impact of the point mutants on transcript levels of genes and operons.

The CodY-regulated genes that are overexpressed in the null mutant but not at all in any of the point mutants include those that encode aconitase (*acn*) and isocitrate dehydrogenase (*icd*) (Fig 9K). The simplest interpretation of this result is that such genes are so strongly repressed by CodY that they are only expressed when CodY is almost totally inactivated. This would occur when the concentrations of the BCAAs and/or GTP in the cell become too low to maintain the minimal concentration of ligand-bound CodY needed to bind to the relevant CodY targets. An extension of this interpretation is that, in cells that are consuming the nutrients that supply ILV and other amino acids, the Krebs Cycle genes would be among the last of the CodY regulon components to be turned on.

**Fig 10:**
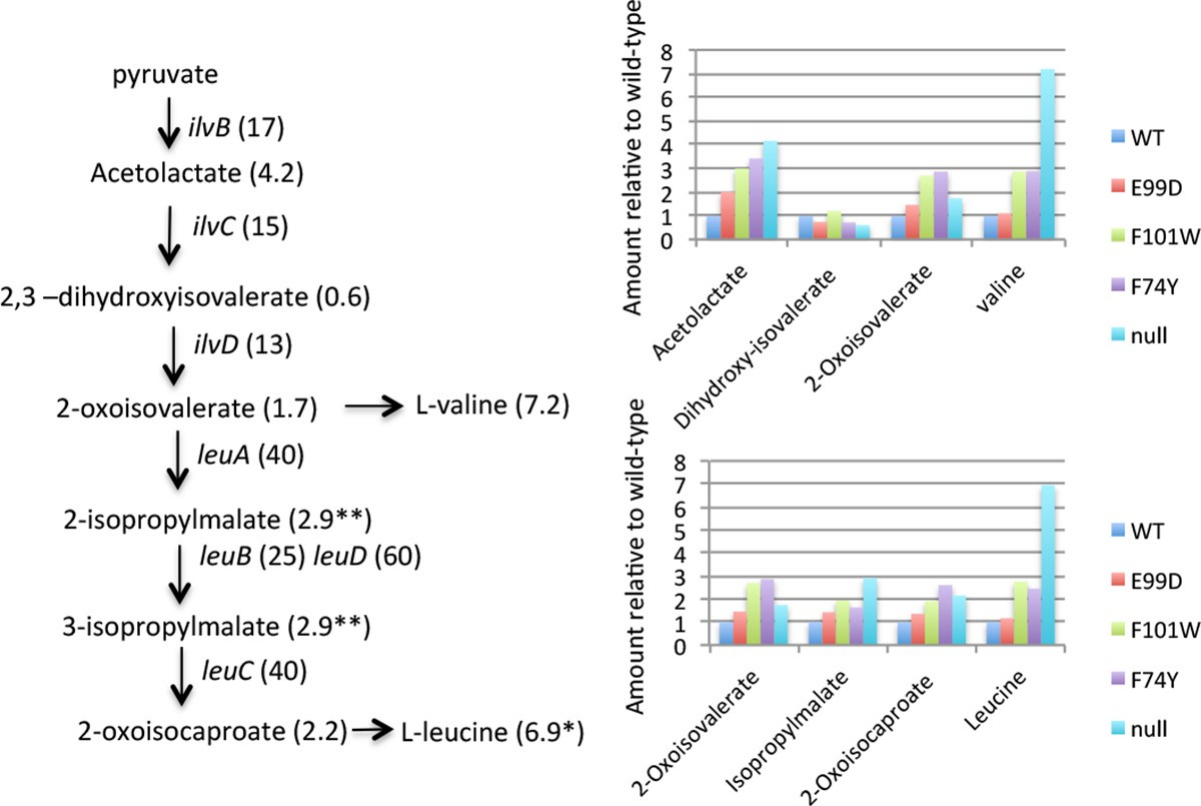
Impact of *codY* mutations on expression of the genes and biochemical intermediates of the BCAA biosynthesis pathway. On the left side of the figure, the pathway from pyruvate to valine and leucine is shown. The genes that code for the various enzymes are in italics. The numbers in parentheses indicate the RPKMO ratios for each of the genes in the *codY* null mutant relative to wild-type and the concentrations of the various metabolites in the *codY* null mutant relative to the wild-type. The right side of the figure shows the results of metabolomics analysis for a panel of *codY* mutants relative to wild-type (the latter set at 1.0 for all metabolites). The pathway to valine is shown above the pathway to leucine. The asterisk associated with the concentration of leucine indicates that the concentration is actually the sum of leucine and isoleucine, which were not separated in this analysis. The double asterisk for 2- and 3-isopropylmalate indicates that 2.9 is the sum of the two inseparable isomers.

This hypothesis is complicated, however. As the intracellular levels of ILV and/or GTP drop, repression by CodY of the ILV biosynthetic pathway and ILV transporters is relieved, resulting in at least partial restoration of the intracellular ILV concentrations. This assumption was tested by analysis of the intracellular concentrations of valine, leucine and intermediates in their biosynthesis. (Intermediates in isoleucine biosynthesis were not analyzed.) As shown in Fig 10, the accumulation of valine, leucine and most intermediates in their biosynthesis in the panel of *codY* mutant cells is consistent with the increased expression of the biosynthesis genes as CodY activity decreases. Note that the measurement of isopropylmalate does not distinguish between 2-isopropyl- and 3-isopropyl- and that the leucine value includes both leucine and isoleucine. The accumulation of amino acids is presumably a result of derepression of both the biosynthesis operons and transporters.

### CodY-mediated regulation of virulence genes

**Fig 11:**
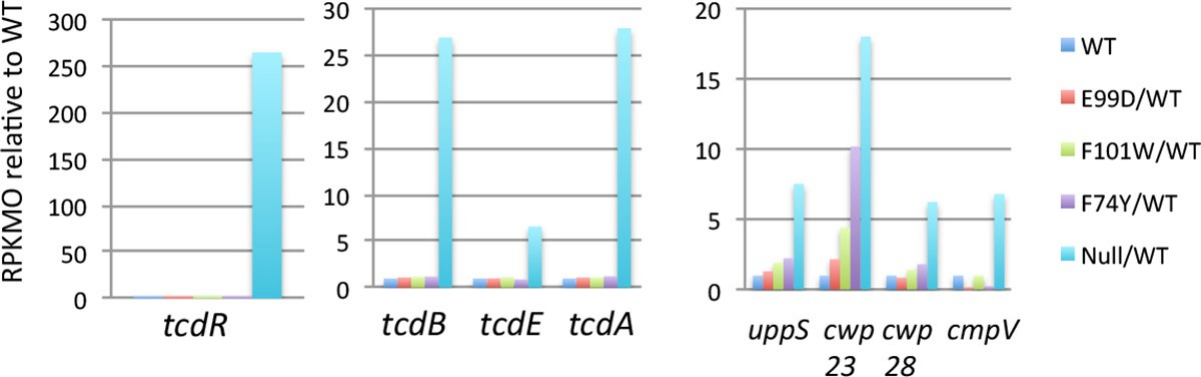
RNA-seq analysis of virulence genes. The relative transcript levels (RPKMO) of eight virulence-associated genes in the *codY* null mutant and three *codY* point mutants is shown. All genes displayed here were overexpressed >3-fold in the *codY* null mutant. The different colored patterns for each gene indicate the relative transcript levels in each of the mutant strains. In part A, the genes of the major toxin locus are shown in their genetic order. In part B, other virulence genes are grouped, but are not genetically linked to each other.

The vast hyperexpression of the major toxin genes (*tcdA*, *tcdB*) in exponential phase cells of the *codY* null mutant of strain UK1 (Fig 11) is consistent with its increased virulence in mice (Fig 1), although other factors must be involved as well. Many other genes are known to contribute to virulence [52]. For instance, the genes encoding the ADP-ribosylating toxin CdtAB were overexpressed 2.5–3.0 fold in the *codY* null mutant, even though the expression of CdtR, the positive regulator of *cdtAB* [53, 54], was not affected by *codY* mutations. Interestingly, the *tcdA* and *tcdB* genes are positively regulated by CdtR in two ribtoype 027 strains [55]. In contrast, transcription of other virulence factor genes, such as the *dlt* operon [56], *fbpA* [57], *groEL* [58], *fliC* and *fliD* [59], the *pdaV-sigV* gene cluster [60], zinc- and collagen-binding protein genes [61–63], and a lipoprotein gene [64] was not affected substantially by a *codY* null mutation. In a cluster of 39 surface protein-related genes that includes *uppS*, *tuaG*, *manC*, *pgm2*, *mviN*, *cwp84*, *cwp66*, *secA2* and *slpA* [65–67], only the *uppS* gene was significantly derepressed (7.5-fold) in a *codY* null mutant (Fig 11). Of 22 other surface protein-encoding genes (Cwp8-Cwp29) [65], the genes encoding CwpV (R20291_0440), Cwp23 (R20291_1698) and Cwp28 (R20291_1911) were derepressed 6.8-, 18.0- and 6.2-fold, respectively, in the *codY* null mutant (Fig 11).

The intracellular concentration of c-di-GMP also has an impact on *C*. *difficile* virulence. Overexpression of the di-GMP cyclase gene CD630_14200 causes a decrease in motility [68] and in expression of the *tcdA* and *tcdB* genes [69]. This effect appears to be due to inactivation by c-di-GMP of SigD, the RNA polymerase sigma factor that activates flagellar genes and the *tcdR* gene [13, 69]. Of the 31 genes encoding di-GMP cyclases or c-di-GMP phosphodiesterases in strain R20291 [70], only one, R20291_0884 (corresponding to CD630_10280), encoding a di-GMP cyclase, was overexpressed (15.9-fold) in the *codY* null mutant of strain UK1 (S4 Table). None of these genes was underexpressed in the *codY* null mutant. Since overexpression of a di-GMP cyclase would be expected to limit virulence, the effect of CodY on R20291_0884 is unlikely to contribute to the *codY* mutant strain’s hypervirulence. Interestingly, a di-c-GMP hydrolase gene, CD630-15150, is repressed by CodY in strain 630 [15, 71], but not in UK1 (R20291_1364; S3 Table).

### Complex regulation of the ethanolamine utilization and other pathways

**Fig 12:**
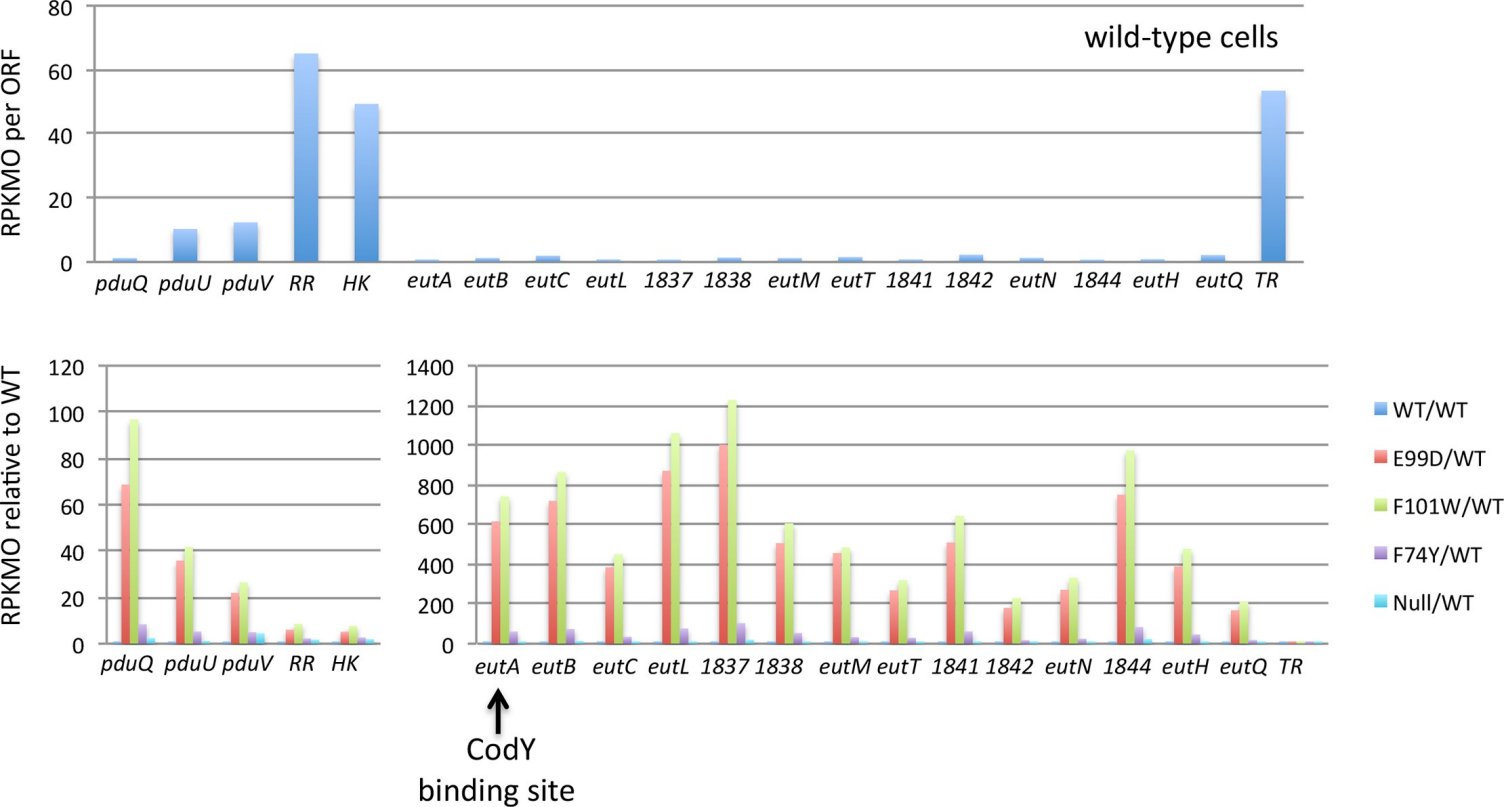
RNA-seq analysis of ethanolamine metabolism gene cluster. Some CodY-repressed genes had unusual patterns of expression in the panel of *codY* point mutants. Shown here are the genes of the ethanolamine metabolism gene cluster, R20291_1828 to 1846. In the top panel, the average RPKMO values for each gene in the wild-type strain are shown. In the bottom panel, the transcript levels in the mutants relative to the wild-type (set at 1.0) are presented. The different colored patterns for each gene indicate the relative transcript levels in each of the mutant strains. The top panel indicates that several genes, including those encoding a putative histidine kinase (HK) and response regulator (RR) are expressed at a relatively high level in the parent strain. The lower panel shows that all genes were overexpressed to a greater extent in two point mutants, E99D and F101W, than in the null mutant.

Although the *codY* point mutant strains had intermediate residual CodY activity with respect to the parent strain and the *codY* null mutant for more than 390 genes (Fig 8), some genes were most highly expressed in the point mutants. A major example is the large gene cluster that encodes the proteins of ethanolamine metabolism (CDR20291_1828–1846) (Fig 12 and S8 Table). In *C*. *difficile* strain 630Δerm, all of these genes are expressed at a high level at 2 hrs after the end of exponential growth [72]. Most of the genes in the *eut* cluster were expressed at so low a level in the *codY* null mutant that they were not included among the 495 genes whose transcripts are increased >3-fold by the null mutation (S3 Table). All the genes of this cluster, however, proved to be very highly expressed in all three *codY* point mutants (S8 Table). In addition, 7 genes, including the *ilvCB* operon (CDR20291_1412–1414) and a cellobiose transport operon (CDR20291_2927–2928), were much more highly expressed in the F101W mutant than in the *codY* null mutant.

The mechanism responsible for the unusual effect of the *codY* point mutations is not known, but the general pattern is similar to that seen for the *B*. *subtilis braB* gene [73]. CodY is a direct repressor of *braB*, but is also a repressor of *scoC*, which encodes a second repressor of *braB*. Thus, as CodY activity diminishes, direct repression of *braB* is initially relieved, but a further decrease in CodY activity leads to ScoC accumulation and restoration of repression. The identities of the putative second regulators of *eut*, *ilvCB* and cellobiose genes are unknown. In the case of *eut*, two genes within the cluster (R20291_1831 and 1832) appear to encode a response regulator and histidine kinase, but they are likely to be positive regulators of *eut* gene expression. An apparent transcription factor encoded immediately downstream of the *eut* gene cluster (R20291_1847) is also unlikely to be the predicted *eut* regulator, since its own expression is only slightly affected by *codY* mutations.

### Regulation by CodY of sporulation gene expression

Endospore formation is a type of bacterial differentiation that is restricted to a handful of bacterial genera; it has been studied principally in the genera *Bacillus* and *Clostridium* and a few of their close relatives. When faced with certain kinds of nutrient limitation, cells of spore-forming species stop dividing and create two compartments within the same cytoplasmic membrane. One compartment, the forespore, is the precursor of the mature spore and is surrounded by the mother cell. The forespore and mother cell express a large number of compartment-specific genes. When the developing spore has reached maturity, the mother cell lyses, releasing the spore into the environment. Both the initiation and the various stages of spore formation are dependent on specific transcriptional regulators. Five different RNA polymerase sigma factors, σ^H^, σ^F^, σ^E^, σ^G^, and σ^K^, are needed for transcription of key genes, some of which are transcribed before internal cell division and others in the separate compartments [74, 75]. The Spo0A protein is a positive regulator of a large number of genes at the onset of sporulation; its own synthesis depends on σ^H^ and it activates transcription of the genes that encode σ^F^ and σ^E^ [76–79].

**Fig 13:**
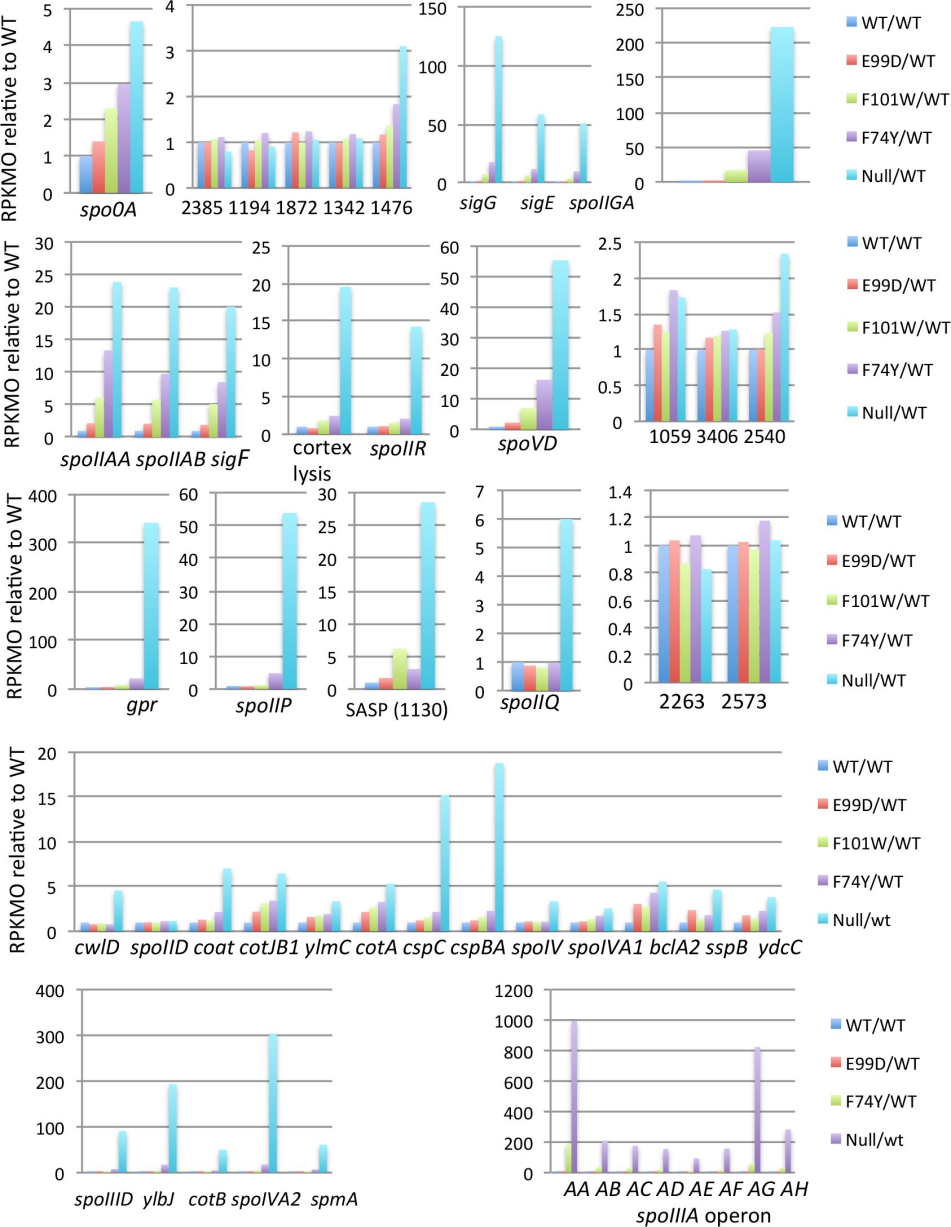
RNA-seq analysis of Spo0A-, σ^F^- or σ^E^-dependent genes. (A-H) Expression patterns of Spo0A-dependent (A-H), σ^F^-dependent (I-M) and σ^E^-dependent (N-P) genes and operons in a panel of *codY* mutants. Transcript levels (RPKMO values) are presented relative to the levels in the wild-type strain (set at 1.0). The different colored patterns within each part of the figure reflect the behavior of individual genes. In parts C, E, F, and P, the genes are from individual clusters. In parts B, H, M, N and O, the genes presented together are not genetically linked. In part P, the expression of the *spoIIIA* operon in strain ND-CD17 was not included, because the transposon carrying the mutant *codY* gene inserted upstream of *spoIIIAA* and reduced expression of the entire operon.

**Fig 14:**
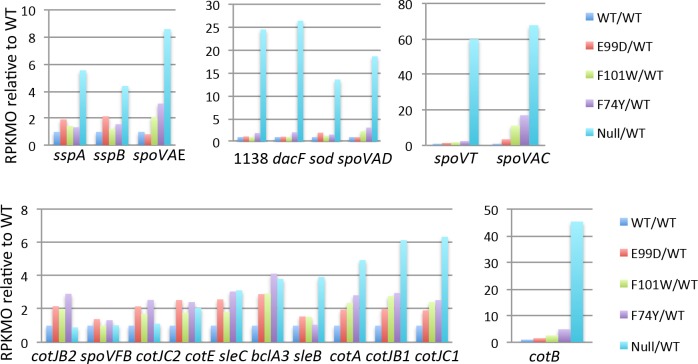
RNA-seq analysis of sporulation genes dependent on σ^G^ or σ^K^. Expression patterns of individual σ^G^-dependent (A-C) and σ^K^-dependent (D-E) genes in a panel of *codY* mutants. Transcript levels (RPKMO values) are presented relative to the levels in the wild-type strain (set at 1.0). The different colored patterns within each part of the figure reflect the behavior of individual genes. Part E displays the results with a single gene (*cotB*), but all other parts of the figure show collections of unlinked genes that have similar levels of overexpression in the *codY* null mutant.

Previous work by Nawrocki *et al*. [17] showed that spore formation and the expression of *spo0A* and the genes that encode sporulation sigma factors σ^F^, σ^E^ and σ^G^ are significantly enhanced in the *codY* null mutant (LB-CD16) of strain UK1 compared to wild-type. The effect of the *codY* mutation was overcome by complementation with the wild-type gene [17]. Consistent with this finding, the *spo0A* gene, which encodes the primary factor needed for initiation of sporulation gene expression, was derepressed during exponential growth phase in the *codY* null mutant (Fig 13A), as was a gene encoding a histidine kinase known to activate Spo0A by phosphorylation (R20291_1476, equivalent to CD630_15790) [80] (Fig 13B). Four other genes encoding potential Spo0A kinases were not affected by *codY* mutations (Fig 13B). The genes and operons encoding σ^F^, σ^Ε^ανδσ^Γ^ were also overexpressed >3-fold in the *codY* null mutant (Fig 13C–13E), as were more than 40 other genes that are required for *C*. *difficile* sporulation or are homologous to known sporulation genes in other bacteria (Table 3 and Fig 13D–13H). The distribution of genes under the control of the various sporulation sigma factors is shown in Fig 13I–13M for σ^F^, Fig 13N–13P for σ^Ε^, Fig 14A–14C for σ^G^ and Fig 14D and 14E for σ^K^. The gene encoding σ^H^ was only overexpressed 1.8-fold (adjusted P value = 0.054) in the *codY* null mutant and the gene encoding σ^K^ was only overexpressed 2.9-fold; neither gene was included in the list of CodY-regulated *spo* genes (Table 3). Moreover, genes encoding quorum-sensing factors that potentially activate Spo0A phosphorylation (R20291_2639–2640 and 3187–3189) [81] were neither overexpressed nor underexpressed more than 3-fold in the *codY* null mutant. Fimlaid et al. [82] reported that in strain JIR8094 more than 200 stationary phase genes depend on Spo0A for their expression. The number of Spo0A-dependent genes in strain JIR8094 was later increased to 434 (A. Shen, personal communication) based on RNA-seq results of Pishdadian et al. [83]. Taking advantage of a table provided by L. Barquist and R. Fagan that matches R20291 genes with those of strain 630, we found that 159 of the 434 Spo0A-dependent genes were overexpressed ≥ 3-fold during exponential growth in the *codY* null mutant (S7 and S8 Tables). Dembek et al. [84] used transposon mutagenesis to identify R20291 genes essential for efficient sporulation. Of the 798 genes in which transposon insertions limited spore formation 4-fold or more, 138 were overexpressed during exponential phase growth in the *codY* null mutant and 14 were underexpressed.

**Table 3:** Hyperexpression of *spo* genes in a *codY* null mutant.

Gene	Expression ratio (*codY* null/WT)	Gene	Expression ratio (*codY* null/WT)
*spo0A*	4.7	*spoIVA*	304
R20291_1476 (Spo0A kinase)	3.5	R20291_0714 (stage IV sporulation protein)	5.1
*spoIIA* operon: *spoIIAA*	24	R20291_3400 (spore cortex-lytic enzyme)	21
*spoIIAB*	23	*sspA*	5.7
*sigF*	20	*sspB*	4.7
*spoIIE*	223	*cspBA*	19
*spoIIG* operon: *spoIIGA*	51	*cspC*	15
*sigE*	59	*gerG*	50
*spoIIP*	61	*spoVAC*	76
*spoIIR*	16	*spoVAD*	21
*sigG*	125	*spoVAE*	9.4
*spoIIIA* operon: *spoIIIAA*	995	*spoVD*	60
*spoIIIAB*	212	*spoVS*	3.2
*spoIIIAC*	177	*spoVT*	670
*spoIIIAD*	157	spore coat assembly	50
*spoIIIAE*	95	R20291_0212 (spore coat protein)	7
*spoIIIAF*	160	*cotJB1*	6.5
*spoIIIAG*	824	*cotJC1*	6.6
*spoIIIAH*	284	*spmA*	61
*spoIIID*	91	*spmB*	14
*sleB*	4.6	*cwlD*	4.6
*sleC*	3.3	*bclA2*	5.6
*gpr*	384	*bclA3*	4.1
		*gerS*	3.8

The indicated genes have been shown to be required for spore formation in *C*. *difficile* or are homologs of *B*. *subtilis* sporulation genes. The expression ratios are based on RPKMO levels as determined by RNA-Seq.

The expression patterns of sporulation operons in the point mutant strains suggested that the level of CodY activity needed to maintain repression of sporulation genes is variable (Figs 13 and 14). The *spo0A* gene was partially activated in all of the point mutants, whereas other sporulation genes required greater inactivation of CodY in order to be expressed, implying either that the level of *spo0A* derepression determines to different extents the level of expression of these genes or that some or all of these genes are also direct targets of CodY.

## Discussion

### Impact of CodY on metabolism

More than 250 of the 552 genes overexpressed or underexpressed more than 3-fold in the *codY* null mutant strain during exponential growth are involved in metabolic processes, e.g., transport and metabolism of carbon and nitrogen sources, biosynthesis of amino acids, energy production, glycogen biosynthesis, sugar metabolism, and the Krebs Cycle. The results obtained with strains expressing mutant forms of CodY with different levels of residual activity imply that some metabolism genes (e.g., the *leu* and *glg* operons) are considerably derepressed when CodY activity is only slightly reduced, some genes (e.g., the *ntp* and *etf* operons) are only significantly derepressed when CodY activity is more reduced and other genes (e.g., the *xdhA* and *aksA* clusters) are only derepressed when CodY activity is eliminated. The implication of these findings is that the affinity of CodY for its binding sites varies considerably from target to target, a situation proven to be true on a genome-wide basis for *B*. *subtilis* CodY [30], but not yet tested for *C*. *difficile* CodY. An apparent implication of these results is that, as cells consume critical nutrients, expression of the operons cited above would increase first for the *leu* and *glg* operons and lastly for the *xdhA* and *aksA* operons. However, this implication may not be correct, because all of these operons are undoubtedly regulated by other factors as well, each of which responds to different changes in nutrient availability. Thus, the timing of expression of CodY-regulated genes and operons when cells transition from exponential to stationary phases is likely to be complex.

### Impact of CodY on virulence

The CodY proteins of multiple Gram-positive pathogens serve as virulence regulators. As shown in Fig 1, despite the high level of virulence of *C*. *difficile* strain UK1, inactivation of CodY further increases its virulence significantly. Ribotype 027 strains have often been viewed as “hypervirulent” compared to other isolates, although not all members of the ribotype are highly virulent [85]. In *S*. *aureus* [39–42, 86, 87] and *Bacillus thuringiensis* [88], CodY is also a negative regulator of virulence. In fact, a hypervirulent *S*. *aureus* USA300 strain became even more virulent when the *codY* gene was inactivated [39]. Thus, the virulence of multiple species is restrained to a significant extent under conditions where CodY is active. In *Bacillus anthracis* [28], *Bacillus cereus* [35], *Listeria monocytogenes* [36], *Clostridium botulinum* [43, 89] and several Streptococcal species [33, 90, 91], however, CodY appears to activate virulence. Moreover, in *Clostridium perfringens*, the impact of CodY on virulence varies from strain to strain. In both the Type D strain CN3718 and the Type A strain SM101, CodY activates virulence gene expression, but the mechanisms of regulation are different. In CN3718, CodY directly activates expression of the *etx* gene, which encodes epsilon toxin [34], whereas, in SM101, CodY activates expression of the *cpe* (*C*. *perfringens* enterotoxin) gene indirectly by positively regulating certain key sporulation genes [92]. By contrast, CodY represses sporulation gene expression in strain CN3718 [34]. The variability in the impact of CodY on virulence and sporulation implies that different strains and species have evolved to use this global regulator in ways that suit their preferred conditions for sporulating and causing damage to the host.

### Mechanism of regulation of sporulation genes by CodY

Virtually all of the genes of known or predicted functions that are expected to be required for spore formation in *C*. *difficile* are expresssed during exponential growth phase in mutant strains defective in CodY. The *spo0A* gene, which encodes the primary regulator of sporulation, is derepressed 4.5-fold in the *codY* null mutant strain (Fig 13). At first approximation, this result is surprising, since many other sporulation genes, all of which depend on direct or indirect activation by Spo0A, are derepressed to a much greater extent. Two factors may be responsible for this level of *spo0A* gene regulation. First, the *spo0A* gene in strain JIR8094 does not have a CodY-binding site [15]; the sequence upstream of *spo0A* in stain UK1 is very similar to that in JIR8094 and presumably also lacks a binding site. Thus, CodY regulation of *spo0A* is undoubtedly indirect. Second, in wild-type cells of strain UK1, *spo0A* mRNA is within the top 4% of mRNAs in terms of abundance (RPKMO value); in the *codY* null mutant, the *spo0A* mRNA moves to the top 3%. Thus, increased synthesis of *spo0A* mRNA may not be critical for spore formation. Instead, phosphorylation of already highly abundant Spo0A is likely to be the critical factor that initiates sporulation. The gene encoding a kinase known to phosphorylate Spo0A *in vitro* [80] has a CodY-binding site [17] and is derepressed in the *codY* null mutant (Fig 13), potentially serving as the primary factor in leading to sporulation gene expression. (The 4.5-fold increase in *spo0A* mRNA in the *codY* null mutant may be due to autoactivation of *spo0A* transcription by Spo0A~P.) In fact, very few sporulation genes have direct CodY-binding sites, suggesting that CodY regulates sporulation genes primarily through indirect effects.

Despite the limited direct effects, the impact of CodY on spore formation by *C*. *difficile* is much greater than for *B*. *subtilis*. Inactivation of CodY increases spore formation by *B*. *subtilis*, but the effect is relatively small and only occurs in particular media [21]. It is likely that the complex regulation of spore formation in *B*. *subtilis* minimizes the impact of inactivation of a single negative regulatory protein. *C*. *difficile*, by contrast, lacks several of the regulators of *B*. *subtilis* spore formation, including Spo0F and Spo0B (members of the Spo0A phosphorylation cascade), Rap proteins that dephosphorylate Spo0F~P, and Spo0E (a Spo0A~P phosphatase). The lack of such complex regulation may explain why a *codY* mutation has a stronger effect on sporulation in *C*. *difficile* than in *B*. *subtilis*.

### Co-regulation of sporulation and toxin synthesis

Toxin synthesis and spore formation are both major contributors to *C*. *difficile* infection. TcdA and TcdB are the primary factors causing damage to the host and spore formation is critical for resistance to antibiotics, for survival of the bacteria outside the GI tract and for transmission of the bacteria to other hosts. Thus, it makes sense that both processes are induced when nutrients become limiting and are subject to overlapping regulation, as shown here and in previous publications [16, 17]. Unlike sporulation genes, which appear to be mostly indirect targets of CodY, the *tcd* locus is a direct target of CodY [16]. Although CodY represses both processes, the results with the point mutants described here imply that many sporulation genes are turned on when CodY is only partially inactivated, whereas expression of the toxin genes requires complete inactivation of CodY.

A potential complication is the likely impact of toxin synthesis on spore formation and *vice versa*. Toxin activity is expected to increase the availability of nutrients due to damage to host cells. In cells that have not yet committed to sporulation, an increase in nutrient availability may permit renewed growth, thereby delaying or preventing sporulation. Since different RNA polymerase sigma factors are required for transcription of toxin genes (σ^A^, σ^D^ and TcdR) [9, 11–13] and sporulation genes (σ^A^, σ^H^, σ^F^, σ^E^, σ^G^ and σ^K^) [74, 76, 82], it seems unlikely that sporulating cells express the toxin genes, but no experiments to date have addressed this issue. How does the *C*. *difficile* population balance the production of toxins and spores? Is the population divided between the two responses to nutrient limitation? The relative behavior of the CodY point mutants described here implies that, as nutrients become limiting, many sporulation genes are turned on; the toxin genes by contrast are among the last genes to be turned on as nutrient availability decreases, implying that at least a portion of the population initiates sporulation before any of the cells produce the toxins. Since spore formation is irreversible after an early stage, the cells that choose to sporulate will complete the process, creating a mixed population that provides multiple advantages to the bacterium. The non-sporulating cells that produce toxin will generate nutrients that will allow them to multiply, while the sporulating members of the population will become prepared to survive outside the GI tract and initiate new infections.

Multiple factors contribute to the regulation of toxin synthesis and sporulation, in some cases providing co-regulation and in other cases opposing regulation. Like CodY, the glucose-activated CcpA protein is a negative regulator of both the *tcd* gene cluster and the *spo0A* and *sigF* genes [14]. In addition, in strain R20291, a *tcdR* mutation causes a decrease in spore formation and heat resistance of the spores produced [10], implying that TcdR is a positive regulator of sporulation as well as toxin synthesis. Possible mechanisms include stimulation of sporulation by toxins (by an unknown mechanism) or a requirement for the TcdR sigma factor for maximal transcription of one or more sporulation genes. On the other hand, in strain 630Δerm, the RstA protein is an activator of sporulation, but an inhibitor of toxin synthesis [93]. In the same strain, one group found that a *spo0A* mutation causes overexpression of the *tcdA* gene [8]. Other researchers detected binding of Spo0A to the *tcdB* gene *in vitro*, but saw little or no effect of a *spo0A* mutation on toxin production *in vivo* [6]. A third group found evidence of overexpression of both *tcdA* and *tcdB* in a *spo0A* mutant of a ribotype 027 strain, but not in 630Δerm [7]. (It is likely that differences in the isolates and media used for these experiments contribute to their varying results.) Although the details remain to be resolved, it is likely that *C*. *difficile* has evolved to base its extent of toxin synthesis and sporulation (and potentially the decision as to which pathway to pursue at the single-cell level) on multiple environmental factors and regulatory proteins.

## Materials and methods

### Bacterial strains and growth conditions

The bacterial strains and plasmids used in this study are listed in Table 4. *C*. *difficile* strains were grown in tryptose-yeast extract (TY) medium [94], BHIS medium [95] or in defined CDMM [96], supplemented with 250 μg D-cycloserine per ml, 40 μg kanamycin per ml, 20 μg thiamphenicol per ml, and 20 μg lincomycin per ml or 5 μg erythromycin per ml, as needed. *C*. *difficile* strains were maintained at 37°C in an anaerobic chamber (Coy Laboratory Products) in an atmosphere of 10% H_2_, 5% CO_2_ and 85% N_2_. *Escherichia coli* and *B*. *subtilis* strains were grown at 37°C in L broth [97] supplemented with 20 μg chloramphenicol per ml or 100 μg ampicillin per ml for *E*. *coli* and with 1 μg of erythromycin and 12.5 μg of lincomycin per ml for *B*. *subtilis*, as needed.

### Containment practices and biosafety precautions

Laboratory experiments using *C*. *difficile* strain UK1 and its derivatives were approved by the Tufts University Institutional Biosafety Committee as registration number 2013-BRIA16 from April 25, 2013 to April 1, 2016 and as number 2016-BR04 from March 31, 2016 to March 31, 2019. All researchers were required to pass annual laboratory safety training tests. In accordance with the registration, live samples of *C*. *difficile* strains were grown and stored in an anaerobic chamber and killed by treatment with bleach or by autoclaving before disposal. To extract DNA, RNA or protein from *C*. *difficile* cultures, the cells were killed in order to allow the molecules to be purified outside the chamber. Researchers handled the material outside the chamber in sealed containers and were protected by gloves and lab coats.

### Storage and sharing plan

Individual strains of *C*. *difficile* were stored on labeled Petri plates inside the anaerobic chamber or at -80°C in tubes containing labels that reveal their origin. The sites of storage were only accessible by researchers who had passed the laboratory safety training tests. If strains of *C*. *difficile* were sent to other labs as part of this project or upon requests by other scientific groups, the bacteria were only sent if the proposed recipient was experienced in working with *C*. *difficile* and dealing with its potential hazards. Such shipments met the requirements of UN 3373 (Biological Substance, Category B, as defined by IATA DGR 6.2 –Infectious Substances). Infection of mice by *C*. *difficile* was carried out under the prescribed, careful conditions described below.

### Strain and plasmid construction

Oligonucleotides used in this study are listed in S8 Table. Creation by TargeTron methodology of an insertional mutation in the *codY* gene of *C*. *difficile* strain UK1 was described by Mooyottu et al. [44]. To complement the *codY* gene disruption, a 983-bp fragment containing the wild-type *codY* gene and its upstream region was amplified using primers oLB275 and oLB276 (S10 Table) and cloned between the *Bam*HI and *HindIII* sites of pBL26, a region homologous to the transposon Tn*916*. The resulting plasmid was named pND3. To create each desired point mutation in the *codY* GAF domain, two sets of primers were designed (S10 Table) to amplify overlapping portions of the *codY* gene, each of which would have the desired mutation. After amplification by Hi-Fidelity Phusion polymerase (New England Biolabs), the two PCR products were purified, mixed and amplified using the primers oLB275 and oLB276, which yielded the entire *codY* gene with the intended point mutation. Each of the mutated genes was then cloned in plasmid pBL26, generating plasmids pND5, pND6, pND7, pND11 and pND12.

**Table 4:** Bacterial strains and plasmids.

Strains and Plasmids	Description	Source and/or Reference
Plasmids		
pJS107	TargeTron vector	J. Sorg
pBL100	TargeTron vector	[98]
pBL92	pBL100 *codY*::*intron*::*ermB*	This work
pBL103	pJS107 *codY*::*intron*::*ermB*	This work
pSMB47	*E*. *coli* plasmid carrying part of Tn*916*	[99]
pBL26	Derivative of pSMB47 carrying the *cat* gene from pJIR1456	[17]
pND3	pBL26::*codY*^+^	This work
pND5	pBL26::codY (F74Y)	This work
pND6	pBL26::*codY* (E99D)	This work
pND7	pBL26::*codY* (E103D)	This work
pND9	pBL26::*codY* (F74L)	This work
pND11	pBL26::*codY* (F101W)	This work
pND12	pBL26::*codY* (P102G)	This work
***Escherichia coli***		
HB101 (pRK24)	F^+^ *supE44 hsdS20*(rB^+^mB^+^) *recA13 ara-14 proA2 lacY1 galK2 rpsL20 xyl-5 mtl-1* (Tra^+^ Mob^+^ Amp^r^ Tc^r^)	[95]
***Bacillus subtilis***		
BS49	Contains integrated Tn*916*	[100]
***Clostridoides difficile***		
UK1	Ribotype 027	D. Gerding
LB-CD16	UK1 *codY*::*intron*::*ermB*	[44]
ND-CD5	UK1 *codY*::*intron*::*ermB* Tn*916*	This work
ND-CD6	UK1 *codY*::*intron*::*ermB* Tn*916*::*codY* (E103D)	This work
ND-CD10	UK1 *codY*::*intron*::*ermB* Tn*916*::*codY*^+^	This work
ND-CD12	UK1 *codY*::*intron*::*ermB* Tn*916*::*codY* (F74Y)	This work
ND-CD13	UK1 *codY*::*intron*::*ermB* Tn*916*::*codY* (E99D)	This work
ND-CD17	UK1 *codY*::*intron*::*ermB* Tn*916*::*codY* (F101W)	This work
ND-CD18	UK1 *codY*::*intron*::*ermB* Tn*916*::*codY* (P102G)	This work

Introduction into *E*. *coli* strain JM107 caused the plasmids derived from pBL26 to create concatemers that facilitated subsequent transformation of *B*. *subtilis* strain BS49, which carries Tn*916* within its chromosome; the *codY* gene inserted within the chromosomal Tn*916* by homologous recombination. The various *B*. *subtilis* strains were then mated with *C*. *difficile* strain LB-CD16 (*codY*::*intron*::*erm*), resulting in strains ND-CD10 (*codY*::*intron*::*erm* Tn*916*::*codY*^*+*^), ND-CD12 (*codY*::*intron*::*erm* Tn*916*::*codY* F74Y), ND-CD13 (*codY*::*intron*::*erm* Tn*916*::*codY* E99D), ND-CD17 (*codY*::*intron*::*erm* Tn*916*::*codY* F101W), and ND-CD18 (*codY*::*intron*::*erm* Tn*916*::*codY* P102G), each of which has a full-length copy of the *codY* gene (with or without a point mutation) and a copy interrupted by the intron. The creation of mutant strains of *C*. *difficile* was approved by the Institutional Biosafety Committee for Tufts University and Tufts Medical Center and was assigned the registration number 2016-BR04 for the period 03/31/16-03/31/19.

To determine the number of *codY*-containing Tn*916* insertions per *C*. *difficile* genome, chromosomal DNA was extracted as previously described (72). As a model, DNA extracted from strain UK1 was 5-fold serially diluted (from 5 to 0.016 ng/μl) and used as a template for quantitative PCR of *codY* (primers oND34/oND35) using the Roche SYBR Green I PCR mix and a Roche LightCycler 480 II thermocycler. Reactions were performed in triplicate in a final volume of 20 μl using 1 μl of the serially diluted DNA and each primer at 0.5 μΜ. The threshold cycle value of each dilution was determined. In a base 10 logarithmic graph, the threshold cycle was plotted versus the dilution factor and the data were fitted to a straight line. The correlation coefficient (R2) for the line was 0.99 or greater. This plot was then used as a standard curve for extrapolating the relative concentration levels of the *codY* gene in strain LB-CD16 carrying Tn*916* embedded with various versions of the *codY* gene. The relative concentration value was then used to generate the number of *codY* copies using the website http://cels.uri.edu/gsc/cndna.html. A separate standard curve was generated for the housekeeping gene *rpoA* using oligos oLB273 and oLB274. The number of *codY* copies in each sample was then normalized to that of *rpoA*. Clones that appeared to have a single full-length *codY* copy per chromosome, indicated with an asterisk in S2 Fig, were used in subsequent studies. However, strain ND-CD13 was later found to have two copies of the *codY* gene (S2 Table).

To map the sites of Tn916 insertion, two approaches were taken. First, applying the method of Hava and Camilli [101] chromosomal DNA of each mutant strain was amplified using a Tn*916*-specific primer (oLS9) and a partially randomized primer (ARB1); after 30 cycles, the products were purified using a QiaQuick PCR Purification Kit (Qiagen) and then subjected to a second round of PCR using a second Tn*916*-specific primer (oLS17) and a non-random primer (ARB2) that anneals to products produced by ARB1. After purification, the products of the second round were sequenced using the Tn*916*-specific primer oLS17. The second approach was a verification based on the RNA-seq results. That is, in most cases, the RNA-seq data revealed a junction between the mRNA for a chromosomal gene and one end of Tn*916*, thereby indicating where the transposon had inserted. In all strains except ND-CD13, only one site of Tn916 insertion was detected. In strain ND-CD13, however, three sites were found. Insertions between R20291_ 0447 and _0448 and between R20291_ 1900 and _1901 contained pSMB47 carrying the mutant version of the *codY* gene. The insertion between R20291_0467 and _0468 did not contain pSMB47.

### Determination of CodY stability and abundance by immunoblotting

*C*. *difficile* strains grown to mid-exponential phase in TY medium (6 ml) were collected by centrifugation and stored at -80°C overnight. Cells were washed with 0.5 mL of solution A (50 mM Tris-HCl, 2 mM EDTA, 1 mM DTT), resuspended in 0.5 mL of solution A and disrupted using a Mini BeadBeater (two beatings of 30 sec each at speed 48.) The samples were stored to -80°C for 1 hour, thawed and subjected to a second round of disruption. Unbroken cells and cell debris were removed by centrifugation at 13,000 rpm for 15 min at 4°C. The total protein concentration of the supernatant was determined using the Bio-Rad protein assay reagent; for each sample, 4 μg of total protein was then analyzed by Western blotting as follows. Protein samples were mixed with an equal volume of 2× SDS-PAGE-loading buffer, boiled for 3 min, loaded on 12% polyacrylamide-SDS gels, and subjected to electrophoresis at a constant voltage of 100V for 2 h. Proteins were then electro-transferred to Immobilon-P membranes (Millipore) and immunoblotted using rabbit antibody to CodY (prepared by Biodesign International (21)) diluted 5000-fold in 5% skim milk in 50 mM Tris, 150 mM NaCl, 0.1% Tween 20. Immune reactions were visualized using an enzyme-based chemiluminescence (ECL) kit (ThermoFisher).

### Animal infection conditions

*C*. *difficile* spores were prepared as described (45). Briefly, an overnight culture in BHIS medium was diluted in fresh medium to an optical density at 600 nm of 0.2. A 150-μl portion of this suspension was spread onto 5 ml of BHIS agar in each well of a six-well tissue culture dish. The dish was incubated anaerobically for 4 to 7 days to induce sporulation. The spores were washed off the plates with phosphate-buffered saline (PBS). The spore suspension was then heated at 60°C for 20 min to kill vegetative cells. The spore suspension was stored at 4°C, and the spore concentration was determined by serial dilution and plating on BHIS agar supplemented with 1% sodium taurocholate.

Four groups of 10 C57Bl/6 mice (Jackson Laboratories) were pretreated with a cocktail of five antibiotics designed to generate the following approximate daily doses: kanamycin (40 mg/kg), gentamicin (3.5 mg/kg), colistin (4.2 mg/kg), metronidazole (21.5 mg/kg), and vancomycin (4.5 mg/kg). Antibiotics were administered in sterile drinking water for 3 days, followed by 2 days of sterile drinking water without antibiotics. Mice were then given one dose of clindamycin (20 mg/kg weight) intraperitoneally one day prior to infection by oral gavage with 10^4^ or 10^5^ spores of C. *difficile* strains UK1 or UK1 *codY* (LB-CD16). After infection, mice were monitored daily for weight loss, diarrhea, and mortality. At a dose of 10^5^ spores, one mouse died after exposure to strain UK1 and two mice died after exposure to LB-CD16. At a dose of 10^4^ spores, no mice died after exposure to either strain. A weight loss of 20% required that the mice be euthanized by carbon dioxide asphyxiation followed by cervical dislocation. At the end of each experiment (7–9 days after infection), mice were euthanized using the same methods.

### Ethics statement

Animal studies followed the Guide for the Care and Use of Laboratory Animals of the National Institutes of Health and were approved by the Tufts University Institutional Animal Care and Use Committee under the protocol #G2012-70. All researchers doing animal studies were required to pass Mandatory Animal Care and Use (MACU) training at Tufts University.

### Quantification of toxin by ELISA

*C*. *difficile* strains were grown in BHIS medium in an anaerobic chamber. Samples (1 ml) were collected after 24 hrs of incubation, and were adjusted to an identical OD_650_ to ensure equal concentrations of cells in each sample. Samples were then filtered and culture fluid was added to Costar 96-well microplates that had been coated with 100 μl of anti-TcdA antibody (PCG 4.1; Novus Biologicals) (1 μg/ml) or anti-TcdB antibody (5A8-E11; GeneTex) (10 μg/ml) and incubated overnight in phosphate-buffered saline (PBS) at 4°C. The samples were then blocked for 1 hr with 5% (wt/wt) skim milk diluted in PBS. Standards (TcdA and TcdB purified as previously described [102]) and samples (100 μl) were added to each well in duplicate and the microplates were incubated for 90 min at 25°C. After another set of washes, HRP-chicken anti-*C*. *difficile* toxin A or B (1:5,000 dilution in PBS, Gallus Immunotech) was added to the wells for 30 min at 25°C. A final set of three washes preceded the addition of TMB Microwell Peroxidase Substrate and incubation for 20 min at 25°C in the dark. The reaction was stopped with 2 N H_2_SO_4_. Absorbance was measured using a plate reader at 450 nm, and the ELISA was analyzed spectrophotometrically utilizing BioTek Gen5 Version 2.0 Data Analysis Software.

### Determination of the crystal structure of *C*. *difficile* CodY

See Supplementary Materials and Methods.

### Quantitative reverse transcription-PCR (qRT-PCR) analysis

The levels of *tcdA* and *tcdB* RNA displayed in Fig 3 were assayed in cultures of *C*. *difficile* grown in CDMM medium and harvested after 8 and 24 hrs (i.e., in stationary phase). DNA-free RNA (500 ng), prepared as previously described [16,103], was quantitated by absorbance (A_260_ and A_260_/A_280_ ratio) using a NanoDrop ND-1000 spectrophotometer (Thermo Scientific) and subjected to cDNA synthesis using a QuantiTect Reverse Transcription Kit (Qiagen) following the manufacturer’s recommendation. To control for contamination by DNA, mock cDNA synthesis reactions containing no reverse transcriptase were used as negative controls in subsequent amplifications. Primers for qRT-PCR were designed using the online PrimerQuest tool from Integrated DNA Technologies (http://www.idtdna.com/Scitools/Applications/Primerquest), and amplification efficiencies for each primer set were determined prior to use. cDNA samples were used as templates for quantitative PCR of *rpoA* (defined as that of R20291_0096) (primers oLB273/oLB274), *tcdA* (primers oLB131/oLB132) and *tcdB* (primers oND32/oND33) using Roche SYBR Green I PCR mix and a Roche LightCycler 480 II thermocycler. Reactions were performed in a final volume of 20 μl using 4 μl of cDNA (25 ng) and 1 μM of each primer. Amplification included 45 cycles of the following steps: 10 s at 95°C, 10 s at 53°C, 15 s at 72°C. Results were calculated using the comparative cycle threshold method [104], in which the amount of target mRNA is normalized to that of an internal control transcript (*rpoA*). Reactions were performed in triplicate using cDNA extracted from three biological replicates, and results are presented as the means and standard deviations of the data obtained.

To assess the impact of *codY* mutations on gene expression during growth, three independent cultures of the wild-type, the null mutant and various *codY* point mutant strains were grown in TY medium to a culture density of A_600_ = 0.4–0.6. Purified RNA was analyzed by qRT-PCR using primers (S10 Table) specific for the genes encoding a peptidase (R20291_2712), chloromuconate cycloisomerase (R20291_1235), *ilvC* (R20291_1413), a cell surface protein (R20291_1698), and *glgC* (R20291_0812), with *rpoA* as a standard for comparison.

### RNA-sequencing library construction, sequencing and analysis

RNA was extracted from two independent cultures of the wild-type strain and two independent cultures of each of the mutant *C*. *difficile* strains grown in TY medium to OD_600_ = 0.4 to 0.6 (mid-exponential phase) using the RNeasy kit (Qiagen) and treated with TURBO DNA-free DNase (Ambion). Removal of DNA was considered successful if no PCR product was detected after 30 cycles of amplification using oND52/53 primers specific for genes encoding 16s rRNA. DNA-free RNA quality was assessed using a Bioanalyzer and RNA Pico Chips (both from Agilent Technologies). Only RNA samples with an RNA integrity number >8 were used for library construction. rRNA was depleted from DNA-free RNA preparations using the RiboZero Magnetic kit (Gram-positive kit; Epicentre). mRNAs were fragmented using the NEB Next RNA Fragmentation Module (New England Biolabs) and further assessed using the Bioanalyzer and RNA Pico Chip. Fragmented RNA was purified and concentrated using the RNA Clean & Concentrator-5 kit (Zymo Research Corporation). First-strand cDNA was synthesized using SuperScript III reverse transcriptase (Life Technologies, Inc.); actinomycin D (8 μg) was included in each reaction to prevent spurious second-strand synthesis. The first-strand cDNAs were purified using the RNA Clean & Concentrator-5 kit and subjected to second-strand cDNA synthesis with 13.3 units of DNA polymerase I and 3.3 units each of *E*. *coli* DNA ligase and RNase H using dUTP in place of dTTP in the reaction mixture. The double-stranded cDNAs were blunted using the Quick Blunting Kit (New England Biolabs) and A-tailed using Klenow fragment (3′-to-5′ Exo minus), to which the universal adaptor Olj331/Olj543 (S10 Table) was ligated in a 10:1 (adaptor/fragment) molar ratio. The libraries were purified and size-selected using AMPure XP SPRI beads (Agencourt), as directed, with elution in 1× low TE buffer [10 mM Tris·Cl (pH 8), 0.1 mM EDTA]. Quantifluor dsDNA dye (Promega) was used to quantify double-stranded cDNA and DNA. The second strand was selectively degraded using 1 unit of USER enzyme (New England Biolabs) before library enrichment and barcoding using Phusion HiFi polymerase (New England Biolabs) and oligonucleotides containing unique 6-bp barcodes. Final library size distributions were determined using a fragment analyzer before pooling samples. Samples were loaded into single lanes of a HiSeq 2500 instrument (Illumina) in the Tufts University Genomics Core and sequenced in multiplex (single-end 50-bp reads) using v3 chemistry. Reads were aligned to the reference genome of the *C*. *difficile* ribotype 027 strain R20291 using Burrows–Wheeler Aligner version 5.9 [105]; the genome sequence and gene annotations for this strain were obtained from RefSeq (www.ncbi.nlm.nih.gov/refseq/). The overall read coverage of genomic regions corresponding to features such as ORFs and rRNAs was conducted as described [51, 106]. Differential expression analysis was conducted using DESeq (50). The primary data from the RNA-seq experiments have been uploaded by the NIH Sequence Read Archive for public access as BioProject PRJNA438155, accessible at https://www.ncbi.nlm.nih.gov/sra?linkname=bioproject_sra_all&from_uid=438155 with accession numbers SRX4158084-SRX4158093.

### Metabolomic analysis

Cultures of *C*. *difficile* strain UK1 and mutant derivatives were grown anaerobically in TY medium to mid-exponential phase (A_600_ = 0.5–0.6). A sample of 13 ml was rapidly collected under vacuum on a nitrocellulose membrane (2 m; Millipore). The membrane was quickly washed with 5 ml of PBS, pH 7.5, to remove excess medium and non-cellular material, and then immersed in 1 ml of a mixture of acetonitrile, methanol and water (40:40:20) supplemented with 0.1 M formic acid pre-cooled at -20°C and stored at -80°C.

Metabolites were extracted from duplicate samples and analyzed by LC-MS as described in de Carvalho et al. [107], Pesek et al. [108] and Brinsmade et al. [30]. Data analysis was as outlined in Brinsmade et al. [30].

## Supporting information

S1 File Supplementary materials and methods. (DOCX)

S1 Fig The quaternary structure of the GAF domain of *C*. *difficile* CodY. **A**. The hexamer formed by the six molecules of the asymmetric unit of the CdCodY(1–156) crystals. The view is down the three-fold symmetry axis with the three intersecting 2-fold symmetry axes in the plane of the page. The chains are coloured A (ice blue) B (gold) C (coral) D (blue) E (pink) and F(red). The isoleucine ligands are shown as spheres with carbon, nitrogen and oxygen atoms coloured green, blue and red respectively. **B**. The dimer formed by the GAF domains of CodY from *B*. *sutbilis*. The two chains are coloured gold and blue respectively and the isoleucine effector is shown as spheres. **C**. The A (ice blue) and B (gold) subunits from the CdCodY GAF domain hexamer shown in **A.** It is evident that the these two moleculesare juxtaposed in a very similar manner to the subunits in *B*. *subtilis* CodY, the obvious difference being that helices α1 have been displaced from the AB dimer interface so that they instead pack with neighbouring dimer interfaces in the hexamer. **D**. Superposition of GAF domain dimer of *B*. *subtilis* CodY (white) with a ‘hybrid’ GAF domain dimer of CdCodY (ice blue) formed by substituting the α1 helices of chains A and B with those from chains F (red) and C (coral) respectively. Following least squares superposition of 238 C_α_ atoms from the GAF domain dimers from the two species, the positional rmsΔ is 1.6 Å. The carbon atoms of the effectors are colored green and grey for CdCodY and BsCodY respectively.
(TIF)

S2 Fig Quantification of *codY* copy number in the different mutant strains. DNA was extracted from *C*. *difficile* wild-type and *codY* mutant strains harboring *codY* variants with single amino acid substitutions and quantified by real time PCR (qPCR). The numbers indicate different isolates of the same *codY* variant. Only clones having a single, full-length, uninterrupted copy of *codY*, indicated with an asterisk (*), were used in subsequent studies.
(TIF)

S3 Fig Verification of stability of mutant CodY proteins. Crude lysates of *C*. *difficile* strains carrying both a *codY* null mutation and a version of the *codY* gene with a point mutation were assayed by Western blotting using rabbit anti-CodY antibodies. Proteins of each lysate (4 μg) were separated by SDS-PAGE. The proteins were electrotransferred and immunoblotted with a polyclonal CodY antibody. Lane 1 contains purified *B*. *subtilis* CodY protein. Lanes 2–10 display extracts of various *codY* point mutants. (Each one is a derivative of strain LB-CD16 (*codY*::*erm*) in which a point mutant form of *codY* has been integrated into the chromosome.) Lanes 11 and 12 display lysates from the strain LB-CD6 with (lane 11) or without (lane 12) the empty vector pBL26. Lane 13 displays the lysate from wild-type cells.
(TIF)

S1 Table X-ray data collection and refinement statistics. (PDF)

S2 Table Sites of Tn*916* insertion in *codY* mutant strains. The chromosomal sites of Tn916-*codY* insertion were determined by sequencing and by analysis of RNA-seq data. See Materials and Methods for details.
(DOCX)

S3 Table RPKMO values for all genes of the parental strain UK1, the *codY* null mutant and three point mutants. Two samples were assayed and averaged for each strain.
(XLSX)

S4 Table **Genes overexpressed (A) or underexpressed (B) >3-fold in the *codY* null mutant strain.** The average RPKMO values for two samples of strains UK1 (*codY*^+^) and LB-CD16 (*codY::intron::erm*) were determined (columns D and G). The ratios of the LB-CD16/UK1 averages are presented in column H.
(XLSX)

S5 Table **Genes overexpressed (A) or underexpressed (B) >3-fold in the *codY* mutant strain ND-CD13.** The average RPKMO values for two samples of strains UK1 (*codY*^+^) and ND-CD13 (*codY::intron::erm* Tn*916*::*codY* (E99D)) were determined (columns D and G). The ratios of the ND-CD13/UK1 averages are presented in column H.
(XLSX)

S6 Table **Genes overexpressed (A) or underexpressed (B) >3-fold in the *codY* mutant strain ND-CD17.** The average RPKMO values for two samples of strains UK1 (*codY*^+^) and ND-CD17 (*codY::intron::erm* Tn*916*::*codY* (F101W)) were determined (columns D and G). The ratios of the ND-CD17/UK1 averages are presented in column H.
(XLSX)

S7 Table **Genes overexpressed (A) or underexpressed (B) >3-fold in the *codY* mutant strain ND-CD12.** The average RPKMO values for two samples of strains UK1 (*codY*^+^) and ND-CD12 (*codY::intron::erm* Tn*916*::*codY* (F74Y)) were determined (columns D and G). The ratios of the ND-CD12/UK1 averages are presented in column H.
(XLSX)

S8 Table Expression of ethanolamine metabolism genes in *codY* mutant strains. The average RPKMO values for each of the genes is shown for the wild-type and the *codY* mutant strains.
(XLSX)

S9 Table Sporulation genes regulated by CodY. CodY-repressed genes (>3-fold) in strain UK1 (annotated as R20291 genes) that were found to be Spo0A-dependent in strain JIR8074, a derivative of strain 630 (A. Shen, personal communication) are listed with their 630 analogs.
(XLSX)

S10 Table List of oligonucleotides. (XLS)
