# Supplementary material for: Impact of CodY protein on metabolism, sporulation and virulence in Clostridioides difficile ribotype 027
Source: PLoS One. 2019 Jan 30;14(1):e0206896. doi: 10.1371/journal.pone.0206896 (PMC6353076; doi:10.1371/journal.pone.0206896)
Supplement: S1 File — (DOCX) [file pone.0206896.s001.docx]

**Supplementary Materials and Methods**

**Cloning for Overexpression:** A DNA fragment encoding residues 1-156 of *C. difficile* CodY (CdCodY) was amplified by the polymerase chain reaction using the oligonucleotides 5'-CCAGGGACCAGCAATGGCAAGTGAAGTGTTACAAAAA-3' and 5'-GAGGAGAAG CGGCGTTATAATATTTCAAGACCAACAACAG-3' with plasmid pEAV1 DNA (1) as template. Using the In-Fusion cloning method (Clontech), this fragment was inserted into the plasmid vector pET-YSBLIC3C, a derivative of pYSBLIC (2), into which a sequence encoding a human rhinovirus 3C cleavage recognition site has been inserted between the hexahistidine codons and the ATG start codon.

Recombinant protein was produced in *E. coli* BL21(DE3). Cells were grown with shaking at 37° C in Luria-Bertani broth containing 30 μg.ml^-1^ kanamycin to an OD_600_ of 0.6-0.8 and induced by addition of 1 mM isopropyl β-D-thiogalactopyranoside and incubation at 16° for 20 hours. Cells were harvested by centrifugation and resuspended in buffer A (50 mM Tris-HCl, pH 7.5, 500 mM NaCl, 10 mM imidazole) in the presence of a protease inhibitor cocktail and lysed by sonication on ice. The soluble cell extract was collected following centrifugation and CdCodY(1-156) was purified in 3-steps. First, the soluble cell supernatant was loaded onto a 5-ml nickel-charged His-Trap column (Amersham Pharmacia) equilibrated in buffer A. After washing, the column was developed with a 10-500 mM imidazole gradient in Buffer A. Fractions containing CdCodY(1-156) were identified and pooled. At this stage, a proportion of the recombinant CdCodY(1-156) protein irreversibly precipitated. The fractions were clarified by centrifugation and the supernatant (2.5 mg.ml^-1^ in protein concentration) was treated with a 1:50 ratio of HRV 3C protease to cleave off the N-terminal tag with simultaneous dialysis overnight against buffer B (20 mM Tris-HCl, pH 7.5, 150 mM NaCl). The cleavage products were passed over a second Ni-NTA-agarose column equilibrated in buffer B. In this step, highly purified untagged protein was identified in the flow-through fractions, which were combined, concentrated by centrifugal ultrafiltration (Amicon Ultra), and passed down a Superdex S200 column in buffer B. After gel filtration, the molecular mass of the purified protein was measured by electrospray ionisation mass spectrometry as 17,339 Da - within 1 Da of the calculated mass of CdCodY(1-156) with an N-terminal Gly-Pro-Ala sequence, representing a vestige of the cloning and proteolysis procedure.

**Crystallisation**: For crystallization experiments, the pooled CdCodY(1-156)-containing fractions were concentrated to 13 mg.ml^-1^ in the presence of 15 mM isoleucine. Multiple preliminary crystallisation conditions were established in vapour diffusion experiments set up in sitting nanodrop format with the PACT and Hampton I and II screens. The crystals and the quality of their diffraction were improved through further manual and robotic optimizations, the use of seeding techniques, and the exploitation of different cryo-protectants. This led to well-diffracting crystals, the best of which were obtained from hanging drop vapour diffusion experiments with a well solution containing 20% polyethylene glycol 3350, 0.2 M sodium formate, 0.1 M bis-Tris-propane, pH 6.5 and protein solutions containing 10 mM isoleucine.

**Structure Determination:** The CodY crystals were cryo-protected using precipitant solution containing 15% (v/v) glycerol and subsequently flash-cooled in liquid nitrogen for data collection. X-ray diffraction data were collected from a single crystal to 1.68 Å resolution on beamline i02 at the Diamond Light Source (DLS) and the data processed, scaled and reduced using the programs XDS (3) and AIMLESS (4). The crystal belonged to space group P2_1_2_1_2, with unit-cell parameters *a* = 120.89 Å, *b* = 190.39 Å, *c* = 43.51 Å *α = β = γ =* 90.0°, with six protein chains in the asymmetric unit.

The structure was solved by molecular replacement with MOLREP (5) and using the GAF domain of CodY from *B. subtilis* with bound Ile (PDB: 2B18), which shares 44 % sequence identity with CodY from *C. difficile,* as the search model. The initial solution revealed four molecules. After preliminary refinement, further molecular replacement calculations gave a complete solution with six molecules. The electron density maps were of sufficient quality for model building and the resulting model was then iteratively built using COOT (6) and refinement by using REFMAC (7). The model was refined to a final R_work_ = 16.2 % and R_free_ = 23.0 % with good stereochemistry. Data collection and refinement statistics are given in Table S1. The atomic coordinates and structure factors have been released through the RCSB Protein data bank and can be obtained through the accession code 5N0L (<https://www.rcsb.org/structure/5n0l>).

Figures derived from the crystal structures were made using CCP4mg (8).

The asymmetric unit of the crystal comprises six GAF domains, six isoleucine effectors, and 602 water molecules. There are no breaks in the main chain electron density and residues Met1-Leu156 of all six chains are well-defined. The six GAF domains of the CdCodY asymmetric unit form a hexamer with 32 symmetry (**Figure S1A**). The hexamer is formed by exchange of the N-terminal α-helices between chains. However, as shown in **Figure S1**, pairs of subunits within the hexamer, are closely similar in structure to the dimers of the GAF domain of BsCodY. In the latter, the α1 helices pack against helices α2 and α5 within the same chain. Subsequent association of helices α1 and α5 with the equivalent helices in the partner subunit gives rise to a dimer interface in the GAF domain of BsCodY formed by four α-helices derived from the two chains. In the CdCodY GAF domain structure, this interface is conserved but, as a consequence of helix swapping, the interface is constituted of four helices from four different subunits.

The observation of a CdCodY GAF domain hexamer was unexpected since the GAF domain of CodY from *B. subtilis* is a dimer (**Figure S1B**). The exchange of the α1-helices is an example of 3D domain swapping a well-characterised phenomenon which alters the quaternary but not the tertiary structure of proteins (9). While 3D domain swapping is integral to the quaternary structure, physiological function, and evolution of many oligomeric proteins, it is often an experimental artefact of overproducing domain fragments of proteins and then maintaining the purified proteins at high concentrations such as those needed for crystallisation. Indeed, we have observed artefactual 3D domain swapping in crystals of sporulation proteins from *B. subtilis* (10, 11).

The elution profile of CdCodY(1-156) during purification by Superdex 200 chromatography was consistent with dimers. Later analysis of the protein by size-exclusion chromatography and multi-angle laser light scattering (SEC-MALLS) revealed two species, an early eluting minor peak associated with a molecular mass of 100 kDa and a later eluting major peak with a mass of 30 kDa consistent with the presence of hexamers and dimers respectively (data not shown). The proportion of hexameric species tended to increase with the age of the protein samples. As stated above, while domain swapping dramatically alters the quaternary structure of proteins, it has little or no effect on the tertiary structure. Since the effector binding pocket of the CdCodY GAF domain is distal to the site of domain swapping, it is unlikely to be affected by it.

**Supplementary figure legends**

**S1 Figure. The quaternary structure of the GAF domain of *C. difficile* CodY.**

**A**. The hexamer formed by the six molecules of the asymmetric unit of the CdCodY(1-156) crystals. The view is down the three-fold symmetry axis with the three intersecting 2-fold symmetry axes in the plane of the page. The chains are coloured A (ice blue) B (gold) C (coral) D (blue) E (pink) and F(red). The isoleucine ligands are shown as spheres with carbon, nitrogen and oxygen atoms coloured green, blue and red respectively. **B**. The dimer formed by the GAF domains of CodY from *B. subtilis*. The two chains are coloured gold and blue respectively and the isoleucine effector is shown as spheres. **C**. The A (ice blue) and B (gold) subunits from the CdCodY GAF domain hexamer shown in **A.** It is evident that the these two molecules are juxtaposed in a very similar manner to the subunits in *B. subtilis* CodY, the obvious difference being that helices α1 have been displaced from the AB dimer interface so that they instead pack with neighbouring dimer interfaces in the hexamer. **D**. Superposition of GAF domain dimer of *B. subtilis* CodY (white) with a ‘hybrid’ GAF domain dimer of CdCodY (ice blue) formed by substituting the α1 helices of chains A and B with those from chains F (red) and C (coral) respectively. Following least squares superposition of 238 C_α_ atoms from the GAF domain dimers from the two species, the positional rmsΔ is 1.6 Å. The carbon atoms of the effectors are colored green and grey for CdCodY and BsCodY respectively.

**S2 Figure.** **Quantification of *codY* copy number in the different mutant strains.** DNA was extracted from *C. difficile* wild-type and *codY* mutant strains harboring *codY* variants with single amino acid substitutions and quantified by real time PCR (qPCR). The numbers indicate different isolates of the same *codY* variant. Only clones having a single, full-length, uninterrupted copy of *codY*, indicated with an asterisk (*), were used in subsequent studies.

**S3 Figure. Verification of stability of mutant CodY proteins.** Crude lysates of *C. difficile* strains carrying both a *codY* null mutation and a version of the *codY* gene with a point mutation were assayed by Western blotting using rabbit anti-CodY antibodies. Proteins of each lysate (4 μg) were separated by SDS-PAGE. The proteins were electrotransferred and immunoblotted with a polyclonal CodY antibody. Lane 1 contains purified *B. subtilis* CodY protein. Lanes 2-10 display extracts of various *codY* point mutants. (Each one is a derivative of strain LB-CD6 (*codY::erm*) in which a point mutant form of *codY* has been integrated into the chromosome.) Lanes 11 and 12 display lysates from the strain LB-CD6 with (lane 11) or without (lane 12) the empty vector pBL26. Lane 13 displays the lysate from wild-type cells.

**Supplementary References**

1. Dineen SS, Villapakkam AC, Nordman JT, Sonenshein AL. Repression of Clostridium difficile toxin gene expression by CodY. Mol Microbiol. 2007;66(1):206-19.

2. Fogg MJ, Wilkinson AJ. Higher-throughput approaches to crystallization and crystal structure determination. Biochem Soc Trans. 2008;36(Pt 4):771-5.

3. Kabsch W. Xds. Acta Crystallogr D Biol Crystallogr. 2010;66(Pt 2):125-32.

4. Evans PR, Murshudov GN. How good are my data and what is the resolution? Acta Crystallogr D Biol Crystallogr. 2013;69(Pt 7):1204-14.

5. Vagin A, Teplyakov A. Molecular replacement with MOLREP. Acta Crystallogr D Biol Crystallogr. 2010;66(Pt 1):22-5.

6. Emsley P, Lohkamp B, Scott WG, Cowtan K. Features and development of Coot. Acta Crystallogr D Biol Crystallogr. 2010;66(Pt 4):486-501.

7. Murshudov GN, Vagin AA, Dodson EJ. Refinement of macromolecular structures by the maximum-likelihood method. Acta Crystallogr D Biol Crystallogr. 1997;53(Pt 3):240-55.

8. McNicholas S, Potterton E, Wilson KS, Noble ME. Presenting your structures: the CCP4mg molecular-graphics software. Acta Crystallogr D Biol Crystallogr. 2011;67(Pt 4):386-94.

9. Bennett MJ, Schlunegger MP, Eisenberg D. 3D domain swapping: a mechanism for oligomer assembly. Protein Sci. 1995;4(12):2455-68.

10. Levdikov VM, Blagova EV, Rawlings AE, Jameson K, Tunaley J, Hart DJ, et al. Structure of the phosphatase domain of the cell fate determinant SpoIIE from Bacillus subtilis. J Mol Biol. 2012;415(2):343-58.

11. Lewis RJ, Muchova K, Brannigan JA, Barak I, Leonard G, Wilkinson AJ. Domain swapping in the sporulation response regulator Spo0A. J Mol Biol. 2000;297(3):757-70.
