# Supplementary figures and images for: Impact of CodY protein on metabolism, sporulation and virulence in Clostridioides difficile ribotype 027
Source: PLoS One. 2019 Jan 30;14(1):e0206896. doi: 10.1371/journal.pone.0206896 (PMC6353076; doi:10.1371/journal.pone.0206896)

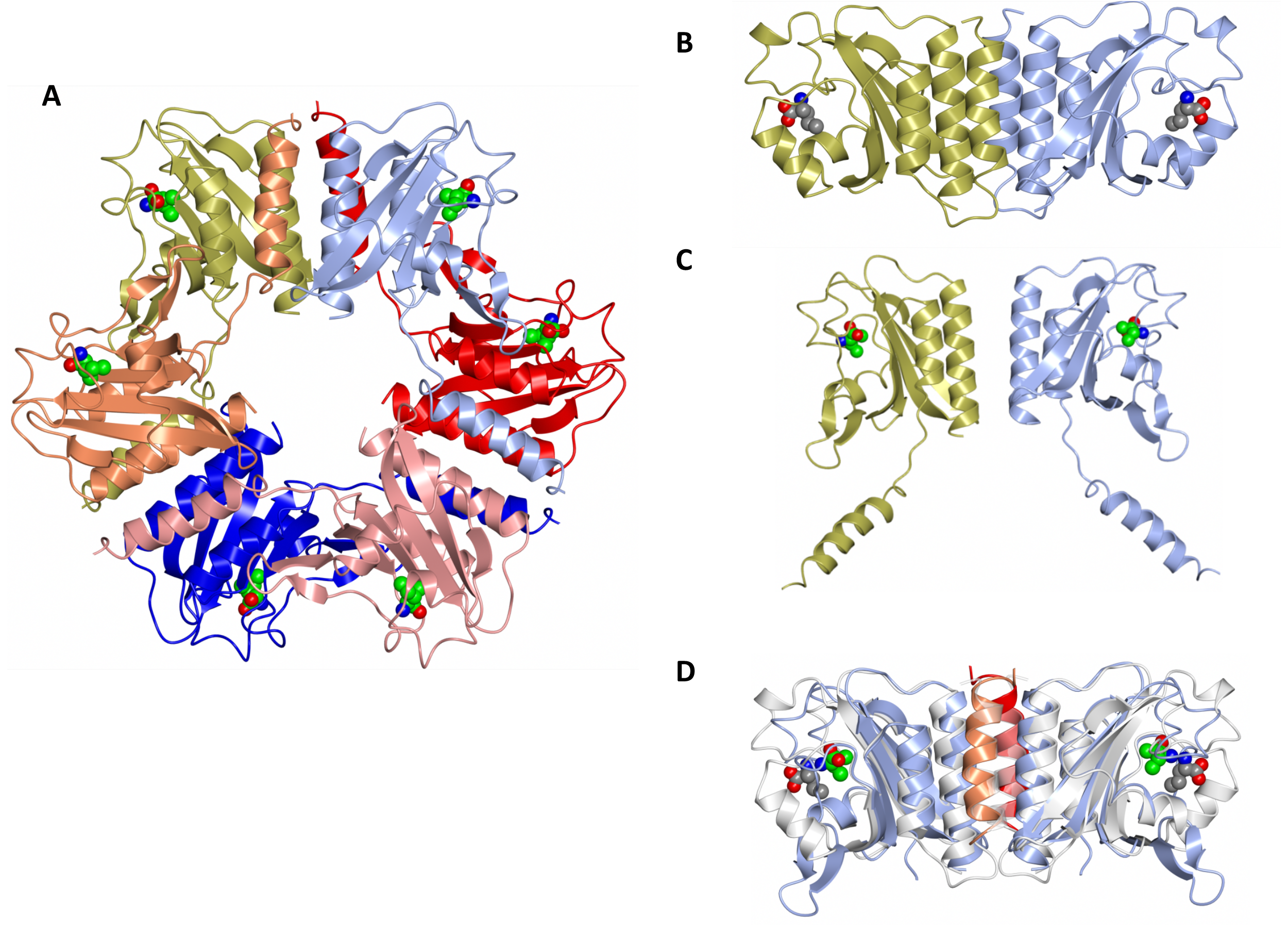

Supplement: S1 Fig — A. The hexamer formed by the six molecules of the asymmetric unit of the CdCodY(1–156) crystals. The view is down the three-fold symmetry axis with the three intersecting 2-fold symmetry axes in the plane of the page. The chains are coloured A (ice blue) B (gold) C (coral) D (blue) E (pink) and F(red). The isoleucine ligands are shown as spheres with carbon, nitrogen and oxygen atoms coloured green, blue and red respectively. B. The dimer formed by the GAF domains of CodY from B. sutbilis. The two chains are coloured gold and blue respectively and the isoleucine effector is shown as spheres. C. The A (ice blue) and B (gold) subunits from the CdCodY GAF domain hexamer shown in A. It is evident that the these two moleculesare juxtaposed in a very similar manner to the subunits in B. subtilis CodY, the obvious difference being that helices α1 have been displaced from the AB dimer interface so that they instead pack with neighbouring dimer interfaces in the hexamer. D. Superposition of GAF domain dimer of B. subtilis CodY (white) with a ‘hybrid’ GAF domain dimer of CdCodY (ice blue) formed by substituting the α1 helices of chains A and B with those from chains F (red) and C (coral) respectively. Following least squares superposition of 238 Cα atoms from the GAF domain dimers from the two species, the positional rmsΔ is 1.6 Å. The carbon atoms of the effectors are colored green and grey for CdCodY and BsCodY respectively. (TIF) [file pone.0206896.s002.tif]

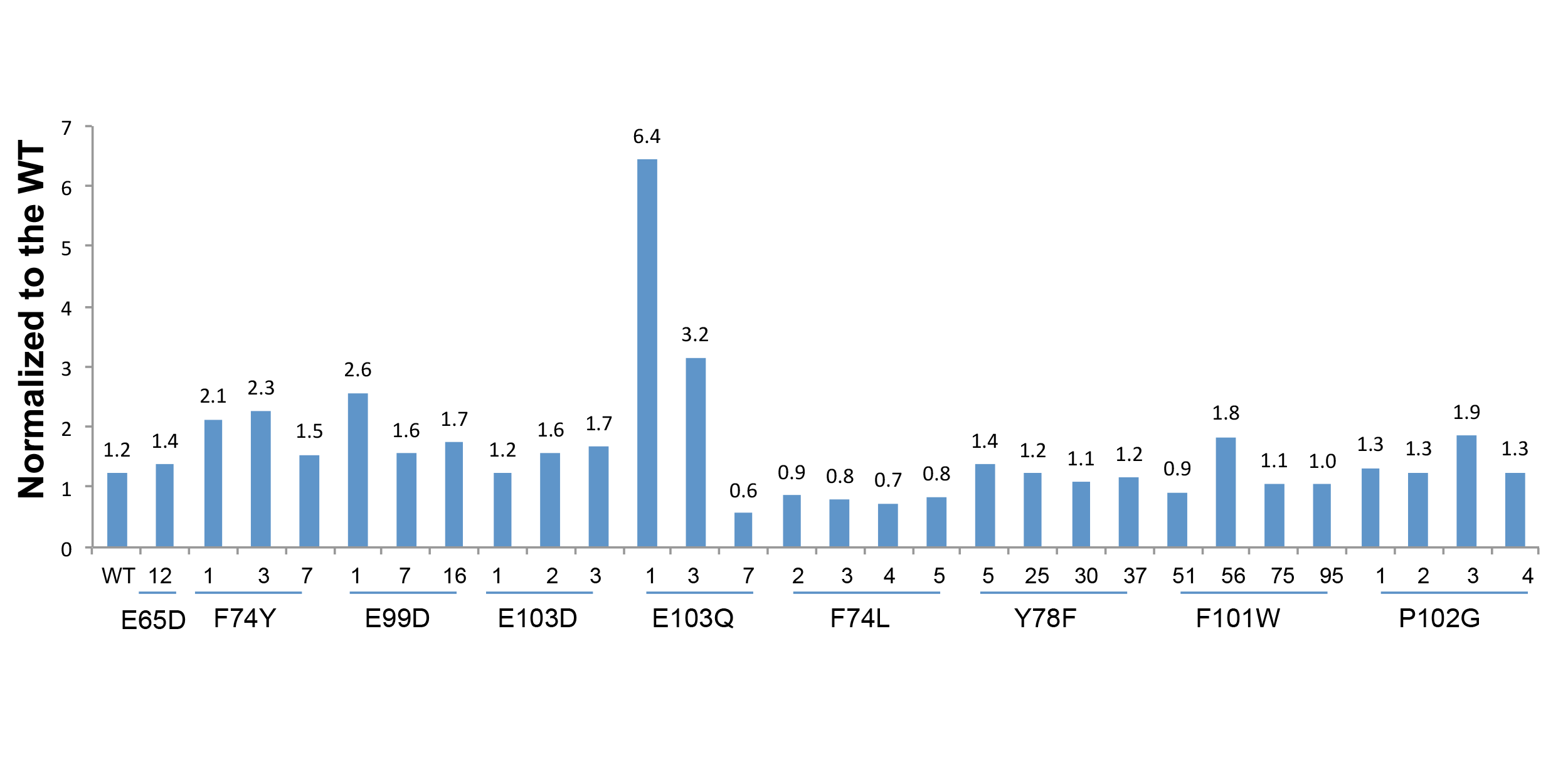

Supplement: S2 Fig — DNA was extracted from C. difficile wild-type and codY mutant strains harboring codY variants with single amino acid substitutions and quantified by real time PCR (qPCR). The numbers indicate different isolates of the same codY variant. Only clones having a single, full-length, uninterrupted copy of codY, indicated with an asterisk (*), were used in subsequent studies. (TIF) [file pone.0206896.s003.tif]

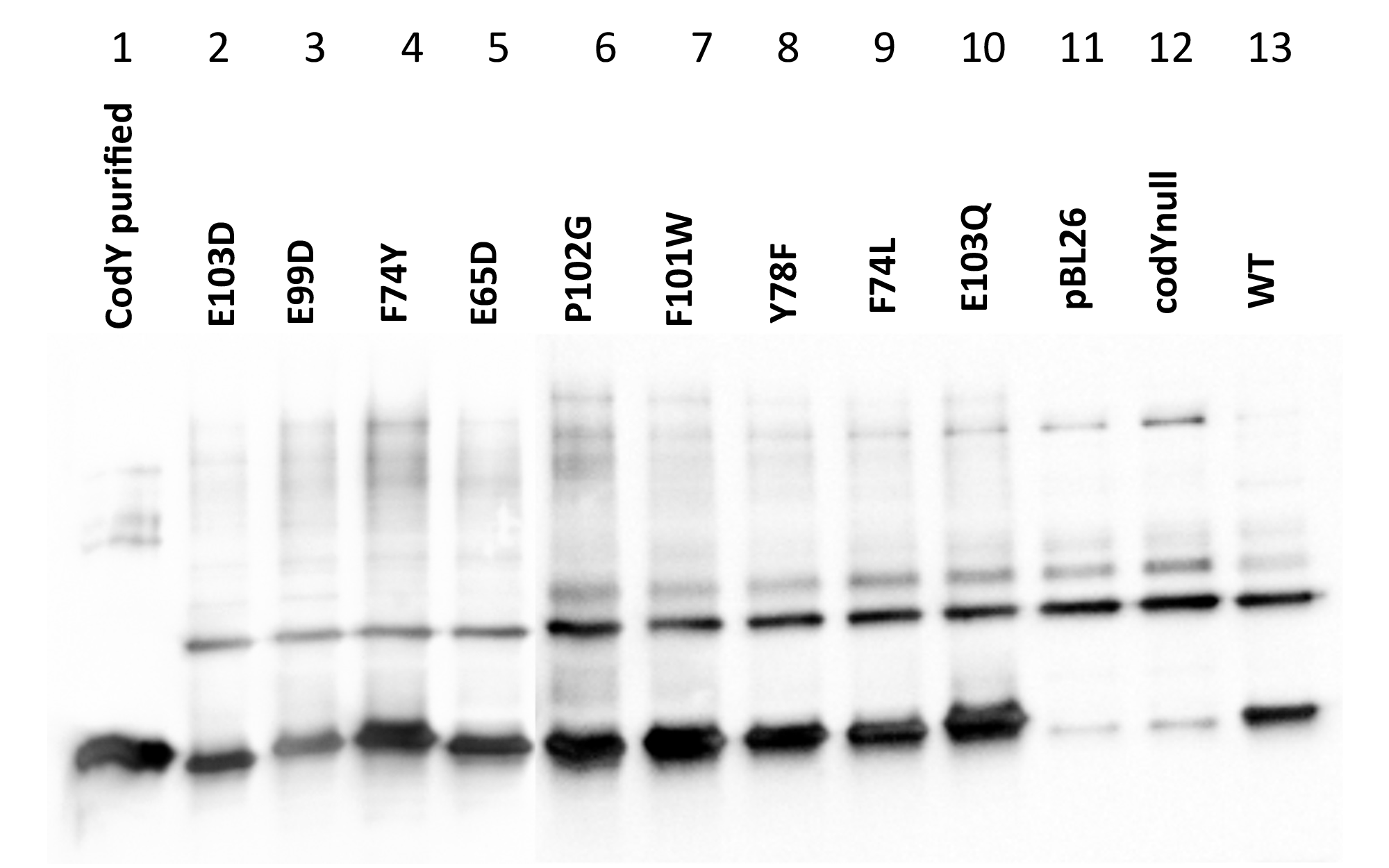

Supplement: S3 Fig — Crude lysates of C. difficile strains carrying both a codY null mutation and a version of the codY gene with a point mutation were assayed by Western blotting using rabbit anti-CodY antibodies. Proteins of each lysate (4 μg) were separated by SDS-PAGE. The proteins were electrotransferred and immunoblotted with a polyclonal CodY antibody. Lane 1 contains purified B. subtilis CodY protein. Lanes 2–10 display extracts of various codY point mutants. (Each one is a derivative of strain LB-CD16 (codY::erm) in which a point mutant form of codY has been integrated into the chromosome.) Lanes 11 and 12 display lysates from the strain LB-CD6 with (lane 11) or without (lane 12) the empty vector pBL26. Lane 13 displays the lysate from wild-type cells. (TIF) [file pone.0206896.s004.tif]
