# Supplementary material for: Impact of CodY protein on metabolism, sporulation and virulence in Clostridioides difficile ribotype 027
Source: PLoS One. 2019 Jan 30;14(1):e0206896. doi: 10.1371/journal.pone.0206896 (PMC6353076; doi:10.1371/journal.pone.0206896)
Supplement: S1 Table — (PDF) [file pone.0206896.s005.pdf]

S1 Table. X-ray Data Collection and Refinement Statistics.

	**CodY *C. difficile* (1-156)** PDB Code 5N0L
Data collection	
X-ray source	DLS beamline i02
Wavelength (Å)	0.97949
Collection Temperature (K)	100
Resolution range (Å)	56.19 - 1.68
Space group	$P2_12_12$
Unit-cell parameters (Å,°)	a = 120.89, b = 190.39, c = 43.51 $\alpha = \beta = \gamma = 90.0$
Matthews coefficient/solvent content (%)	2.44 / 49.6
Number of unique reflections overall/outer shell[a]	115,569 / 8,396
Completeness (%), overall/outer shell[a]	100.0 / 99.9
$I/\sigma(I)$, overall/outer shell[a]	17.1 / 1.2
R_{merge}[b], overall/outer shell[a]	0.067 / 1.814
$CC_{½}$[h]	0.999 / 0.515
Refinement and model statistics	
R-factor[c] (R-free[d])	0.162 (0.230)
Reflections (working/free)	109,833 / 5,726
Outer shell R-factor[c] (R-free[d])	0.312 (0.375)
Outer shell reflections (working/free)	7981 / 408
Molecules/asymmetric unit	6
Number of protein non hydrogen atoms	8,141
R.m.s. deviation from target[f]	
Bond lengths (Å)	0.027
Bond angles (°)	2.808
Average B-factor ($Å^2$)	38.21
Ramachandran plot[g]	96.8/2.9/0.4

[a]The outer shell corresponds to 1.72 – 1.68 Å

[b]$R_{merge} = \sum_{HKL}\sum_I |I_I - \langle I\rangle| / \sum_{HKL}\sum_I \langle I\rangle$ where I_I is the intensity of the ith measurement of a reflection with indexes hkl and $\langle I\rangle$ is the STATISTICALLY WEIGHTED AVERAGE REFLECTION INTENSITY.

[c]R-factor = $\sum||F_o| - |F_o||/\sum|F_o|$ where F_o and F_c are the observed and calculated structure factor amplitudes, respectively.

[d]R-free is the R-factor calculated with 5 % of the reflections chosen at random and omitted from refinement.

[e]Outer shell for refinement corresponds to 1.680 – 1.724 Å

[f]Root-mean-square deviation of bond lengths and bond angles from ideal geometry.

[g]Percentage of residues in preferred regions/ allowed regions/ outliers.

[h]$CC_{1/2}$ is the correlation coefficient between two randomly selected half data sets as described in Karplus &Diederichs (2012)
