# Supplementary material for: Impact of CodY protein on metabolism, sporulation and virulence in Clostridioides difficile ribotype 027
Source: PLoS One. 2019 Jan 30;14(1):e0206896. doi: 10.1371/journal.pone.0206896 (PMC6353076; doi:10.1371/journal.pone.0206896)
Supplement: S2 Table — The chromosomal sites of Tn916-codY insertion were determined by sequencing and by analysis of RNA-seq data. See Materials and Methods for details. (DOCX) [file pone.0206896.s006.docx]

S2 Table. Sites of Tn916 insertion

| Mutation | Location of Tn916  by RNA-seq | Location of Tn916 by sequencing | Gene location |
| --- | --- | --- | --- |
| WT | No site detected | Not tested |  |
| E65D (ND-CD4) | 3543608 | 3543611-3543617 | Within R20291_2977  (transcription antiterminator) |
| E103D (ND-CD6) | 2523073 | 2523071-2523073 | Between R20291_2149 and R20291_2150 |
| F74Y (ND-CD12) | 540204 | 540204-540207 | Between R20291_0447 and R20291_0448 |
| E99D (ND-CD13) | (a) 540204  (b) 2220892  (c) Not detected | (a) ~540206  (b) 2220892  (c) 564837-564838 | (a) Between R20291_0447 and 0448  (b) Between R20291_1900 and 1901  (c) Between R20291_0467 and R20291_0468 |
| F74L (ND-CD15) | 10026 | Not tested | Within *sigB* (accompanied by 4-kb deletion at end of Tn916) |
| F101W (ND-CD17) | No site detected | 1249957-1249959 | Between R20291_1029 and R20291_1030 |
